# 5′tRF‐GlyGCC Promotes Breast Cancer Progression via LDHA‐Mediated Glycolysis and Macrophage Polarization

**DOI:** 10.1002/advs.202514031

**Published:** 2025-11-27

**Authors:** Cheng Yi, Yunqing Lu, Xing Chang, Ying Wang, Jiawang Zhou, Guoyou Xie, Lijun Tao, Zhaotong Wang, Yifan Tian, Lihong Wang, Feng Tang, Yijing Zheng, Xinchen Yue, Jinping Lei, Xiansong Wang, Lichen Ge, Zhuojia Chen, Hongsheng Wang

**Affiliations:** ^1^ Guangdong Provincial Key Laboratory of Chiral Molecule and Drug Discovery State Key Laboratory of Anti‐Infective Drug Discovery and Development School of Pharmaceutical Sciences Sun Yat‐sen University Guangzhou 510006 China; ^2^ State Key Laboratory of Oncology in South China Collaborative Innovation Center for Cancer Medicine Sun Yat‐sen University Cancer Center Guangzhou 510060 China; ^3^ Department of Laboratory Medicine Third Affiliated Hospital of Sun Yat‐sen University Guangzhou 510630 China

**Keywords:** 5′tRF‐GlyGCC, breast cancer, FGFR1, LDHA, metabolic reprogramming

## Abstract

Breast cancer (BC) progression is intricately linked to the dysregulation of transfer RNA‐derived fragments (tRFs). Through comprehensive analysis of The Cancer Genome Atlas (TCGA) data, it is demonstrated that 5′tRF‐GlyGCC is overexpressed in BC tissues and negatively associated with patients' survival. Mechanistically, 5′tRF‐GlyGCC binds to lactate dehydrogenase A (LDHA), enhancing its enzymatic activity and promoting glycolysis, which drives BC cell malignancy. This binding is mediated by the phosphorylation of LDHA at tyrosine 10, and facilitated by fibroblast growth factor receptor 1 (FGFR1), through the formation of a ternary complex that amplifies oncogenic signaling. Furthermore, 5′tRF‐GlyGCC/LDHA axis induces macrophage infiltration and polarization toward an M2 phenotype, mediated by the chemokine CCL7, thereby reshaping the tumor microenvironment. Additionally, it is uncovered that the biogenesis of 5′tRF‐GlyGCC is regulated by ALKBH3 and ANG, which also modulate LDHA activity. In vivo, targeting 5′tRF‐GlyGCC/LDHA signaling significantly suppresses tumor growth and enhances the efficacy of immunotherapy. Collectively, these findings elucidate the pivotal role of 5′tRF‐GlyGCC in BC progression, highlighting its potential as therapeutic target for BC treatment.

## Graphical abstract

5’tRF‐GlyGCC promotes breast cancer malignancy by binding to LDHA. This interaction, facilitated by FGFR1 and LDHA phosphorylation, enhances glycolysis. Additionally, 5’tRF‐GlyGCC/LDHA signaling recruits and polarizes macrophages into a pro‐tumor M2 state via CCL7, thus remodeling the tumor microenvironment. Its production is controlled by ALKBH3 and ANG. Critically, disrupting this axis in vivo inhibits tumor progression and improves immunotherapy response.

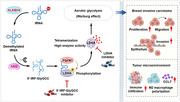

## 1 Introduction

Breast cancer (BC) is the most common cancer affecting women and the second leading cause of cancer‐related death globally.^[^ ^1^ ^]^ Each year, around 1.7 million women are diagnosed with the disease, with over half of these cases occurring in developing countries.^[^ ^1^ ^]^ The need for effective diagnostic and prognostic markers is pressing, yet the molecular and cellular basis of BC remains elusive despite extensive research. Unraveling the mechanisms driving the initiation, progression, and metastasis of BC is thus an imperative scientific challenge. Metabolic reprogramming is a hallmark of tumor cells and a crucial driver of cancer progression.^[^ ^2^, ^3^ ^]^ The Warburg effect is among the most significant metabolic alterations in cancer, with the majority of cancer cells undergoing reprogramming to drive glycolysis, thereby maintaining the energy and biomolecules required for rapid growth and proliferation.^[^ ^3^ ^]^ Therefore, enhanced glycolysis plays a key role in promoting cancer progression, offering new therapeutic targets for BC treatment.^[^ ^2^, ^3^ ^]^

Lactate dehydrogenase A (LDHA) is a key enzyme in glycolysis, catalyzing the conversion of pyruvate to lactate in the final step of the pathway.^[^ ^2^, ^3^ ^]^ Overexpression of LDHA is observed in a variety of cancers, including breast,^[^ ^4^ ^]^ lung,^[^ ^5^ ^]^ prostate,^[^ ^6^ ^]^ pancreatic,^[^ ^7^ ^]^ and liver cancers.^[^ ^8^ ^]^ Aberrantly overexpressed LDHA is a key driver of BC progression. Consistently, accumulating studies demonstrate that targeted inhibition of LDHA—either by suppressing its expression or attenuating its enzymatic activity—can significantly impede BC progression.^[^ ^9^, ^10^, ^11^ ^]^ Notably, LDHA exhibits significant differences in expression and prognostic relevance across the major molecular subtypes of BC. Especially, LDHA is highly expressed in the aggressive triple‐negative BC (TNBC) and human epidermal growth factor receptor 2 (HER2)‐enriched BC subtypes, and its high expression is closely associated with poor prognosis of patients with these two BC subtypes.^[^ ^4^, ^10^, ^12^ ^]^ LDHA can facilitate breast tumor progression by regulating chronic inflammation and immune evasion.^[^ ^13^ ^]^ Recent studies have shown that LDHA promotes the recruitment of immunosuppressive cells and the transformation of tumor‐associated macrophages (TAMs),^[^ ^14^, ^15^ ^]^ highlighting its unrecognized functions within the immune system. For instance, the high expression of LDHA in BC promotes the growth of myeloid‐derived suppressor cells (MDSCs) within the body, thereby suppressing antitumor immunity and the expression of effector T cells.^[^ ^16^ ^]^ Therefore, further investigation into the regulatory factors for LDHA will contribute to a deeper molecular understanding of tumors.

In recent years, transfer RNA‐derived fragments (tRFs) have emerged as important players in cancer development. These fragments are generated continuously under normal and stress conditions by enzymes and proteins like Dicer,^[^ ^17^ ^]^ angiogenin (ANG),^[^ ^18^ ^]^ and RNase Z.^[^ ^19^ ^]^ They include 5′‐tRFs, 3′‐tRFs, and internal tRNA fragments (i‐tRFs), which are produced by enzymatic cleavage at various sites within mature tRNAs.^[^ ^20^ ^]^ Emerging evidence indicates that these molecules exert regulatory effects on core cancer hallmarks, including cell proliferation, invasion, metastasis, as well as gene expression, through diverse mechanisms. These mechanisms encompass degradation of mRNAs, interference with the translational process, and modulation of protein functions.^[^ ^21^, ^22^, ^23^, ^24^ ^]^ For instance, i‐tRFs derived from tRNA‐Glu, tRNA‐Asp, tRNA‐Gly, and tRNA‐Tyr compete with the RNA‐binding protein YBX1 for binding to the 3′UTR regions of various oncogenes, thereby eliminating YBX1's stabilizing effects on their mRNAs. This reduces the production of their oncogenic proteins, ultimately inhibiting BC metastasis.^[^ ^25^ ^]^ Nonetheless, whether metabolic reprogramming and the tumor microenvironment can be modulated by tRFs, and if so, their roles and mechanisms in the occurrence and progression of BC, remain to be elucidated.

This study reveals that 5′tRF‐GlyGCC, generated via ALKBH3‐mediated demethylation followed by ANG‐dependent cleavage, directly interacts with LDHA and its phosphorylating kinase FGFR1, subsequently promoting LDHA phosphorylation at Tyr10 and tetramer formation to enhance its enzymatic activity. Furthermore, patients with higher expression of 5′tRF‐GlyGCC in BC tissues exhibited shorter survival rates. Therefore, the 5′tRF‐GlyGCC/LDHA axis represents a potential target for BC therapies.

## 2 Results

### 2.1 5′tRF‐GlyGCC Promotes BC Progression by Enhancing Malignant Phenotypes and Metabolic Reprogramming

**Figure 1:**
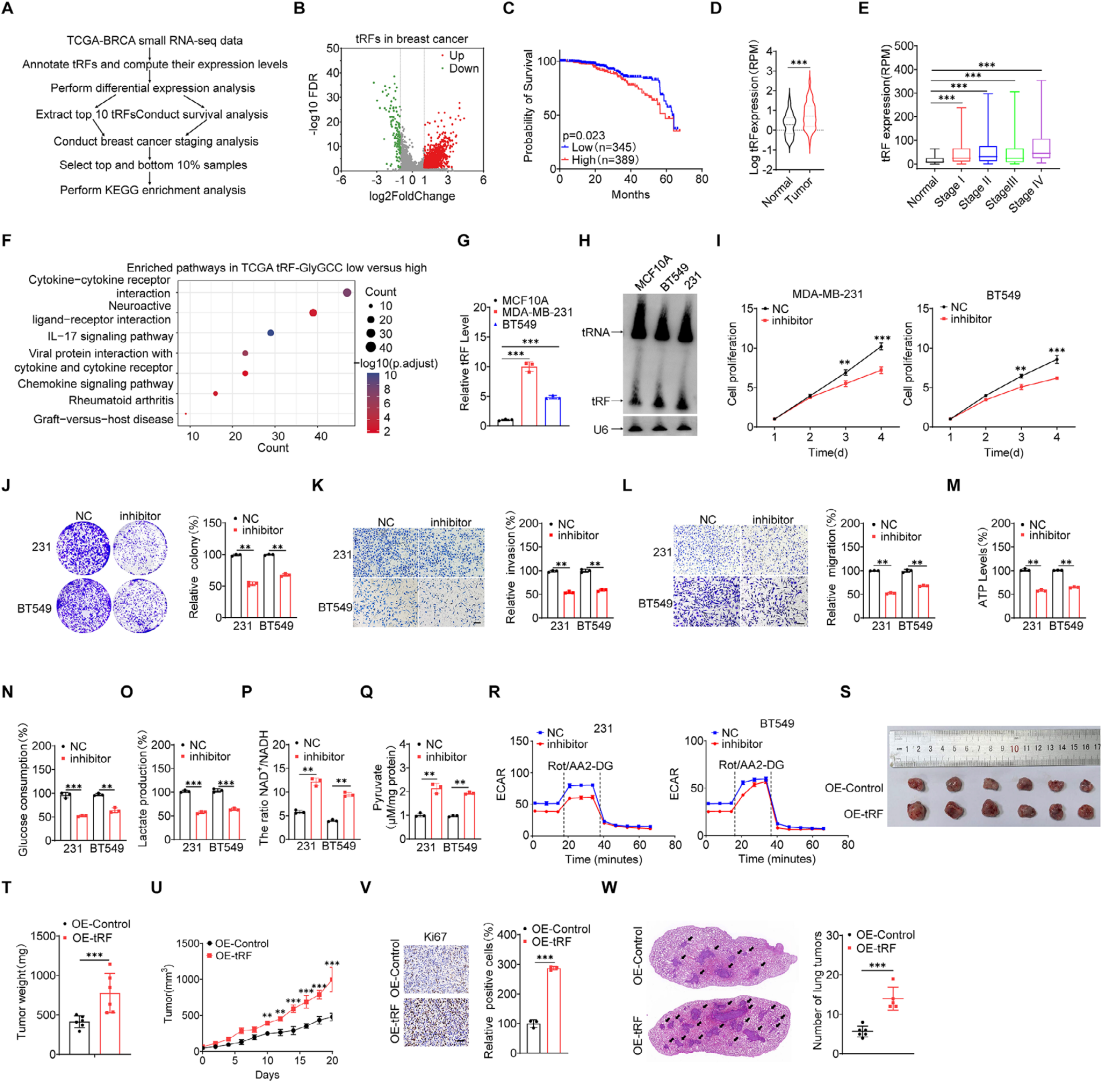
5′tRF‐GlyGCC Promotes BC Progression by Enhancing Malignant Phenotypes and Metabolic Reprogramming. A) Schematic diagram for the workflow for filtering TCGA‐BRCA small RNA‐seq data in the TCGA database. B) Volcano plot of tRFs expression in BC patients from the TCGA database. Red dots indicate significantly upregulated tRFs in BC patients with a log2 fold change (log2FC) greater than 1 and a false discovery rate (FDR) less than 0.05. Green dots indicate significantly downregulated tRFs with a log2FC less than ‐1 and an FDR less than 0.05. Grey dots represent tRFs with no significant change in expression in BC patients. C) Association of 5′tRF‐GlyGCC expression with overall survival of patients in the TCGA‐BRCA cohort. D) Box plots showing the differential expression of tRFs between normal (n = 89) and matched tumor (n = 89) samples in the TCGA‐BRCA cohort. E) Expression of 5′tRF‐GlyGCC in different stages of the TCGA BRCA cohort. Data are presented as box plots. The center line in the box indicates the median, and the box represents the first and third quartiles. *p* values are determined by a two‐tailed Wilcoxon test. Normal, n = 104; StageI, n = 184; Stage II, n = 627; Stage III, n = 248; Stage IV, n = 20. F) KEGG analysis of upregulated genes in 5′tRF‐GlyGCC ‐high compared to 5′tRF‐GlyGCC ‐low patients from the TCGA‐BRCA cohort. Upregulated genes were significantly enriched in the cytokine‐cytokine receptor interaction and chemokine signalling pathways. G, H) Levels of 5′tRF‐GlyGCC in the MCF10A, MDA‐MB‐231, and BT549 cell lines by RT‐qPCR (G) or northern blot (H) analysis. I) The CCK‐8 assay was used to assess the proliferation of BC cell lines following transfection with the 5′tRF‐GlyGCC inhibitor. J) The colony formation assays (left) and their quantitative analysis (right) were utilized to evaluate the clonogenic potential of BC cells 48 h after transfection with the 5′tRF‐GlyGCC inhibitor. K,L) The Transwell assays (left) and their quantitative analysis (right) were conducted to assess changes in the invasive (K) and migratory (L) abilities of BC cells 48 h after transfection with the 5′tRF‐GlyGCC inhibitor, scale bar: 100 µm. M–O) The ATP levels (M), glucose consumption (N), and lactate production (O) assays were performed to evaluate changes in the metabolic profile of BC cells 48 h following transfection with the 5′tRF‐GlyGCC inhibitor. P) The NAD⁺/NADH ratio assay was performed on MDA‐MB‐231 and BT549 cell lines subsequent to their transfection with 5′tRF‐GlyGCC inhibitor. Q) The pyruvate concentration assay was performed on MDA‐MB‐231 and BT549 cell lines, subsequent to their transfection with 5′tRF‐GlyGCC inhibitor. R) The extracellular acidification rate (ECAR) assays were used to detect changes in glycolysis of BC cells 48 h following transfection with the 5′tRF‐GlyGCC inhibitor. S–U) Representative images of mice bearing tumors, derived from the stable overexpression of 5′tRF‐GlyGCC in a xenograft nude mouse model, are shown (S). Tumor weight (T) and tumor volume (U) were assessed. V) IHC was employed to assess(left) and quantitatively analyze (right) the alterations in the Ki67 within the tumor tissue following the overexpression of 5′tRF‐GlyGCC. Scale bars: 100 µm. W) H&E‐stained serial sections from a lung metastasis nude mouse model (left) and quantitatively analyzed (right). H&E; scale bars: 100 µm. Data are presented as mean ± SD from three independent experiments. ^*^ *p* < 0.05, ^**^ *p* < 0.01, ^***^ *p* < 0.001, ns, no significant.

To investigate the tRFs involved in BC progression, we commenced our inquiry by examining a BC small RNA‐seq dataset from The Cancer Genome Atlas (TCGA‐BRCA), which comprised 104 normal and 1103 tumor samples (**Figure** **1**A). Our examination disclosed a notable upregulation of 1168 tRFs and a downregulation of 131 tRFs in BC (Figure 1B). Among the tRFs with increased expression, 5′tRF related to tRNA‐HisGTG, GlyGCC, and GluTTC were the three most significantly upregulated tRFs (Figure S1A, Supporting Information). We also conducted survival analyses on the top ten upregulated tRFs (Table S1, Supporting Information) and within BC using the TCGA database. The findings indicated a significant negative correlation of the patients’ survival with the levels of 5′tRF‐GlyGCC (Figure 1C), but not other tRFs (Figure S1B–J, Supporting Information). Thereafter, we reanalyzed the expression levels of 5′tRF‐GlyGCC in tumor and adjacent samples using TCGA data, revealing a statistically significantly higher expression of 5′tRF‐GlyGCC in tumor samples than adjacent samples (Figure 1D). The data indicated that 5′tRF‐GlyGCC was upregulated in BC tissues and correlated with poor prognosis.

Further, we discovered a significantly positive correlation between 5′tRF‐GlyGCC and proliferation marker PCNA (Figure S1K, Supporting Information), and a significantly negative correlation with tumor suppressor PTEN (Figure S1L, Supporting Information). We also found a significant association between high 5′tRF‐GlyGCC expression and BC staging (Figure 1E), and increased expression of 5′tRF‐GlyGCC was observed in all subtypes of BC compared to normal tissues (Figure S1M, Supporting Information). We selected the top 10% of samples with high 5′tRF‐GlyGCC expression and the bottom 10% with low 5′tRF‐GlyGCC expression for gene differential expression analysis (Figure S1N, Supporting Information). KEGG enrichment analysis of the upregulated genes identified a significant enrichment in the cytokine–cytokine receptor interaction and chemokine signaling pathways (Figure 1F). Consistently, both RT‐qPCR (Figure 1G; Figure S1O, Supporting Information) and Northern blot (Figure 1H; Figure S1P, Supporting Information) analyses showed significantly increased 5′tRF‐GlyGCC levels in the BC cell lines MDA‐MB‐231, BT549, MCF7, and MDA‐MB‐453 compared to the nontumorigenic mammary cell line MCF10A. The data suggest that 5′tRF‐GlyGCC is a pivotal regulator in BC, influencing clinical outcomes and modulating the expression of genes engaged in cytokine signaling.

We then investigated the biological functions of 5′tRF‐GlyGCC in BC. The 5′tRF‐GlyGCC inhibitor significantly suppressed the proliferation (Figure 1I; Figure S1Q, Supporting Information), colony formation (Figure 1J), invasion (Figure 1K), and migration (Figure 1L) of BC cells. Conversely, the 5′tRF‐GlyGCC mimic markedly enhanced these cellular processes (Figure S1R–U, Supporting Information). Additionally, the treatment of 5′tRF‐GlyGCC inhibitor significantly suppressed ATP synthesis (Figure 1M; Figure S1V, Supporting Information), glucose production (Figure 1N; Figure S1W, Supporting Information), and lactate production (Figure 1O; Figure S1X, Supporting Information), but increased the NAD⁺/NADH ratio (Figure 1P) and pyruvate concentration (Figure 1Q) in BC cells. In contrast, 5′tRF‐GlyGCC mimic exerted opposite effects on these metabolic processes (Figure S1Y–AC, Supporting Information). Extracellular acidification rate (ECAR) assay demonstrated that the transfection of 5′tRF‐GlyGCC inhibitor (Figure 1R) and mimic (Figure S1AD, Supporting Information) significantly altered the overall glycolytic flux in BC cells.

Further, stable overexpression of 5′tRF‐GlyGCC in the MDA‐MB‐231 cells led to a significant increase in the size (Figure 1S), weight (Figure 1T), and volume (Figure 1U) of xenograft tumors in nude mice relative to the control group. Immunohistochemistry (IHC) assays revealed that overexpression of 5′tRF‐GlyGCC significantly upregulates Ki67 expression, suggesting that 5′tRF‐GlyGCC overexpression markedly enhanced the cell proliferation of solid tumors in nude mice (Figure 1V). In addition, heightened expression of 5′tRF‐GlyGCC in a previously developed highly metastatic MDA‐MB‐231 cell line in our laboratory^[^ ^26^ ^]^ notably facilitated the lung metastasis of BC, in comparison to the control group (Figure 1W). Collectively, these results elucidate that 5′tRF‐GlyGCC plays a pivotal role in enhancing the malignant phenotypes and metabolic reprogramming of BC cells.

### 2.2 5′tRF‐GlyGCC Drives BC Malignancy by Binding LDHA and Boosting Its Activity

**Figure 2:**
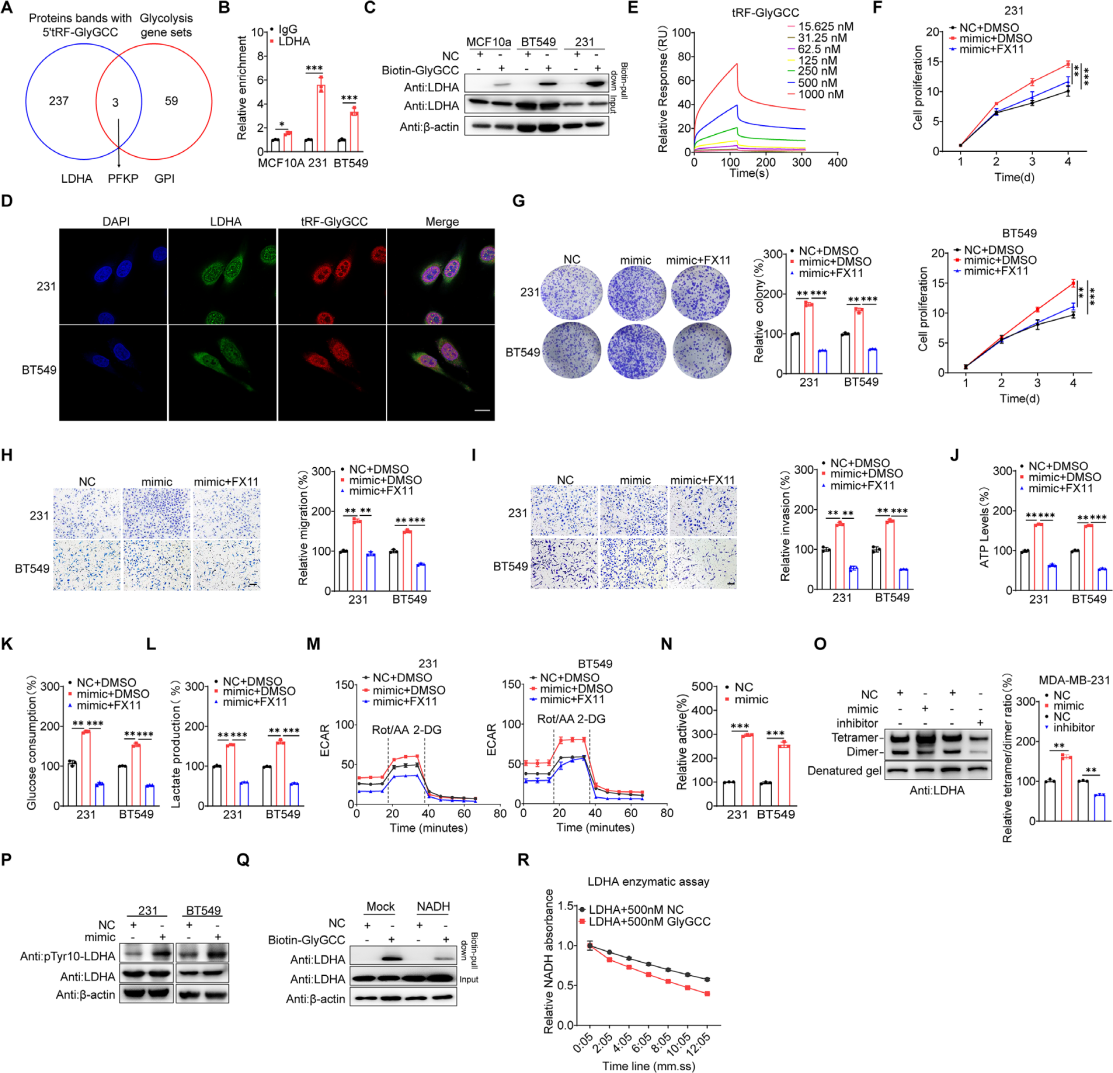
5′tRF‐GlyGCC Drives BC Malignancy by Binding LDHA and Boosting Its Activity. A) Schematic of the RNA‐pulldown assay followed by LC‐MS/MS using a 5′tRF‐GlyGCC sense probe for the identification of proteins that specifically bind 5′tRF‐GlyGCC and then intersect with the Glycolysis pathway of GSEA gene set. B) Association of LDHA with 5′tRF‐GlyGCC in BC cells determined by RIP assays followed by RT‐qPCR. IgG was used as a negative control. C) Western blot analysis of products from RNA‐pulldown assays using 5′tRF‐GlyGCC or a nontargeting oligo suggested 5′tRF‐GlyGCC binding to LDHA protein. D) The cellular localizations of 5′tRF‐GlyGCC and LDHA were analyzed by combining RNA‐FISH and immunofluorescence. Cell nuclei were stained with DAPI, and the scale bar was 20 µm. E) The molecular interaction between LDHA and 5′tRF‐GlyGCC was analyzed to measure the dissociation factor K_d_. F) The relative cell proliferation of MDA‐MB‐231 cells transfected with 5′tRF‐GlyGCC mimic or nontargeting oligo for 48 h, then treated with FX11 20 µm for 72 h. G) The relative cell colony formation assay (left) and quantitatively analyzed (right) of MDA‐MB‐231 and BT549 cells transfected with 5′tRF‐GlyGCC mimic or nontargeting oligo for 48 h, then treated with FX11 20 µm for 24 h, and the. H, I) The relative cell migration (H) and invasion (I) assay (left) and quantitatively analyzed (right) of MDA‐MB‐231 and BT549 cells transfected with 5′tRF‐GlyGCC mimic or nontargeting oligo for 48 h, then treated with FX11 20 µm for 24 h, and the scale bar was 100 µm. J–L) The ATP levels (J), glucose consumption (K), and lactate production (L) assays were performed to evaluate changes in the metabolic profile of BC cells after transfection with the 5′tRF‐GlyGCC mimic or a nontargeting oligo for 48 h, followed by treatment with 20 µm FX11 for 24 h. M) The ECAR assays were used to detect changes in glycolysis of BC cells after transfection with the 5′tRF‐GlyGCC mimic or a nontargeting oligo for 48 h, followed by treatment with 20 µm FX11 for 24 h. N) The activity of endogenous LDHA protein was analyzed in BC cell lines after transfection with 5′tRF‐GlyGCC inhibitor (right). O) Native Western blot analysis was conducted to evaluate (left panel) and quantitatively determine (right panel) the effect of the transfected 5′tRF‐GlyGCC mimic or inhibitor on LDHA tetramer formation in MDA‐MB‐231 cells. P) Western blot analysis was utilized to investigate the effects of transfection with a 5′tRF‐GlyGCC mimic on the phosphorylation of LDHA protein in MDA‐MB‐231 and BT549 cells. Q) Pulldown of LDHA from MDA‐MB‐231 cell lysate expressing 5′tRF‐GlyGCC using Cibacron blue agarose was evaluated by immunoblot. R) Activity of 500 nm recombinant LDHA measured in the presence of increasing amounts of recombinant 5′tRF‐GlyGCC mimic. Data are presented as mean ± SD from three independent experiments. ^*^ *p* < 0.05, ^**^ *p* < 0.01, ^***^ *p* < 0.001, ns, no significant.

To investigate the mechanisms for 5′tRF‐GlyGCC‐triggered BC progression, we analyzed the proteins that interact with 5′tRF‐GlyGCC by conducting RNA immunoprecipitation experiments followed by LC‐MS/MS analysis. This analysis revealed 240 proteins that interact with 5′tRF‐GlyGCC (Table S2, Supporting Information), meeting the criteria of log2FC ≥ 1 and a *p*‐value < 0.05 (**Figure** **2**A). The results showed that 5′tRF‐GlyGCC plays a critical role in enhancing the glycolysis of BC cells. By intersecting the differentially expressed proteins with the glycolysis pathway of the GSEA gene set (Table S3, Supporting Information), we identified LDHA, GPI, and PFKP as common targets in both the 5′tRF‐GlyGCC biotin‐pull‐down protein dataset and the glycolysis gene set (Figure 2A). Meanwhile, we employed a biotin‐labeled RNA pull‐down assay to assess the binding capacity of three glycolytic proteins—LDHA, GPI, and PFKP—to 5′tRF‐GlyGCC, and the results demonstrated that LDHA exhibited the strongest binding capacity to 5′tRF‐GlyGCC (Figure S2A, Supporting Information). Given the critical roles of LDHA in tumor glycolysis and oncogenesis,^[^ ^27^, ^28^ ^]^ and the expression level of LDHA in BC was significantly higher than that of GPI and PFKP (Figure S2B, Supporting Information), we subsequently focused on the roles of LDHA in 5′tRF‐GlyGCC‐induced BC progression.

The RIP assay using an LDHA antibody showed that LDHA could significantly bind 5′tRF‐GlyGCC in BC cells, and this binding in BC cells was much greater than that in MCF10A cells (Figure 2B). Subsequently, RNA biotin pull‐down assays confirmed that LDHA specifically interacts with 5′tRF‐GlyGCC in BC cells (Figure 2C). Further, RNA fluorescent in situ hybridization (RNA‐FISH) combined with immunofluorescence technology showed that the two molecules were co‐localized (Figure 2D). Moreover, surface plasmon resonance (SPR) showed that the LDHA could directly bind to 5′tRF‐GlyGCC, with a dissociation constant K_d_ as 5.93 × 10^−7^ m (Figure 2E). Collectively, these data suggest that 5′tRF‐GlyGCC directly binds to LDHA.

To explore whether LDHA was involved in the oncogenic roles of 5′tRF‐GlyGCC in BC development, we blocked the activity of LDHA using its inhibitor FX11 in cells transfected with 5′tRF‐GlyGCC mimic. FX11 significantly rescued 5′tRF‐GlyGCC‐induced proliferation (Figure 2F), colony formation (Figure 2G), migration (Figure 2H), and invasion (Figure 2I) of BC cells. Concurrently, the increases of ATP synthesis (Figure 2J), glucose production (Figure 2K), and lactic acid production (Figure 2L) caused by 5′tRF‐GlyGCC mimic overexpression were also significantly reduced by the treatment of FX11. Lastly, in ECAR experiments, we observed that the glycolytic capacity and glycolytic reserve, which were promoted by 5′tRF‐GlyGCC mimic, were also significantly reduced after the treatment with FX11 (Figure 2M). These results suggest that LDHA mediates 5′tRF‐GlyGCC‐induced glycolysis and malignant phenotypes of BC cells.

We further investigated the mechanisms responsible for LDHA‐mediated oncogenic roles of 5′tRF‐GlyGCC. The results showed that 5′tRF‐GlyGCC did not affect the expression of LDHA protein (Figure S2C, Supporting Information) or its subcellular distribution within BC cells (Figure S2D, Supporting Information). However, 5′tRF‐GlyGCC mimic significantly enhanced LDHA enzymatic activity in BC cells (Figure 2N), while 5′tRF‐GlyGCC inhibitor significantly suppressed LDHA enzymatic activity in these cell lines (Figure S2E, Supporting Information). Previous studies indicate that the activity of the LDHA enzyme is affected by its tetramerization.^[^ ^29^ ^]^ Our results showed that the formation of LDHA tetramers in nontumorigenic mammary cells was significantly lower compared to the BC cell lines (Figure S2F, Supporting Information). Further, 5′tRF‐GlyGCC mimics facilitated the assembly of LDHA tetramers within BC cells, whereas the administration of 5′tRF‐GlyGCC inhibitors suppressed the tetramerization (Figure 2O; Figure S2G, Supporting Information). LDHA tetramerization is determined by its phosphorylation at tyrosine 10 (pTyr10) mediated by FGFR1.^[^ ^30^ ^]^ Our results demonstrated significantly higher pTyr10‐LDHA levels in the BC cell lines than in the nontumorigenic mammary cells (Figure S2H, Supporting Information). Further, 5′tRF‐GlyGCC promoted the Tyr10 phosphorylation of LDHA in BC cells (Figure 2P). The data indicate that 5′tRF‐GlyGCC boosts LDHA activity via tetramerization and Tyr10 phosphorylation.

NADH association with the LDHA activity is a prerequisite for its substrate binding. We then investigated the effect of 5′tRF‐GlyGCC on the binding of NADH to LDHA. Cibacron Blue 3GA (Cib) is commonly used for affinity purification of enzymes containing NADH cofactor binding sites.^[^ ^31^ ^]^ In BC cells overexpressing 5′tRF‐GlyGCC, the binding of LDHA to Cib‐agarose was greatly reduced, suggesting that 5′tRF‐GlyGCC interfered with the binding of NADH to LDHA (Figure 2Q; Figure S2I, Supporting Information). Further, we incubated the LDHA protein with 5′tRF‐GlyGCC mimic or a scrambled control in vitro using NADH as the substrate. The results demonstrate that 5′tRF‐GlyGCC facilitates the binding of LDHA to its substrate (Figure 2R; Figure S2J, Supporting Information). Collectively, these data indicate that 5′tRF‐GlyGCC can specifically bind LDHA and regulate its tetramerization and activity.

### 2.3 Molecular Insights into 5′tRF‐GlyGCC and LDHA Binding Mechanism

**Figure 3:**
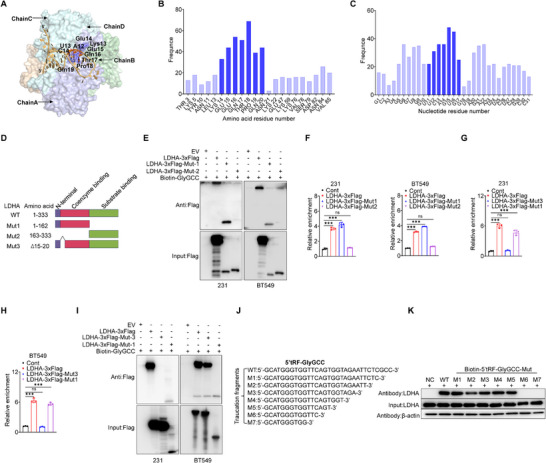
Molecular Insights Into 5′tRF‐GlyGCC and LDHA Binding Mechanism. A–C) The predicted dominant binding mode of 5′tRF‐GlyGCC on LDHA. The LDHA protein is shown on the surface with different colors for different chains, and 5′tRF‐GlyGCC is shown by orange ribbons. The dominant amino acid and nucleotide residues that form hydrogen bond and salt bridge interactions are shown by dark blue and orange–red sticks, respectively. The binding mode is plotted using PyMol (A). The statistical analysis of the amino acid residues on LDHA that form hydrogen bond and salt bridge interactions with 5′tRF‐GlyGCC (B); The statistical analysis of nucleotides that form hydrogen bond and salt bridge interactions with amino acid residues 14–20 on LDHA (C). D) Schematic diagram of LDHA protein mutant segmentation. E) Biotin‐pull down experiments were performed to analyze the binding of different LDHA mutants to 5′tRF‐GlyGCC in MDA‐MB‐231 (left) and BT549 (right) cells. F) RIP experiments were conducted to detect the enrichment of different LDHA mutants with 5′tRF‐GlyGCC, followed by subsequent analysis using RT‐qPCR. G, H) RIP experiments were conducted to detect the enrichment of LDHA‐Mut‐1 and LDHA‐Mut‐3 with 5′tRF‐GlyGCC in MDA‐MB‐231 (G) and BT549 (H) cells, followed by subsequent analysis using RT‐qPCR. I) Biotin‐pull down experiments were performed to analyze the binding of LDHA‐Mut‐1 and LDHA‐Mut‐3 to 5′tRF‐GlyGCC in MDA‐MB‐231 (left) and BT549 (right) cells. J) Schematic diagram of 5′tRF‐GlyGCC mutants. K) Biotin‐pull down was conducted to examine the binding of various 5′tRF‐GlyGCC mutants to LDHA in MDA‐MB‐231 cells. Data are presented as mean ± SD from three independent experiments. ^*^ *p* < 0.05, ^**^ *p* < 0.01, ^***^ *p* < 0.001, ns, no significant.

We further investigated the interaction of 5′tRF‐GlyGCC with LDHA and interrogated its binding sites on LDHA using molecular dynamics (MD) and ensemble docking simulations. The potential binding site and important amino acid residues on LDHA were identified by statistical analysis of the hydrogen bond and salt bridge interactions between LDHA and 5′tRF‐GlyGCC from the ensemble docking simulations (**Figure** **3**A). As shown in Figure 3B and 5’tRF‐GlyGCC mainly binds to the interface of chains A, B, C, D in LDHA and dominantly interacts with amino acids 15–20 on LDHA through hydrogen bond or salt bridge interactions. Furthermore, we statistically analyzed the nucleotide residues that form hydrogen bonds or salt bridges with amino acid residues 15–20 to investigate the primary nucleotide residues of 5′tRF‐GlyGCC that bind with LDHA. The results revealed that U12‐G17 are the main nucleotide residues interacting with the residues 15–20 on LDHA (Figure 3C).

Based on the simulation results, we designed three truncated mutants of LDHA (Figure 3D). To assess the binding affinity between 5′tRF‐GlyGCC and these LDHA mutants, we carried out biotin‐pull‐down assays and observed the marked interaction of 5′tRF‐GlyGCC with the LDHA WT and its Mut‐1, but not Mut‐2 (Figure 3E). These results were corroborated by a Flag antibody‐based RIP assay, which verified the stronger interactions of 5′tRF‐GlyGCC with wild‐type (WT) and Mut‐1 than with Mut‐2 (Figure 3F). Building upon this experimental foundation, we constructed the LDHA truncated mutant LDHA‐Mut‐3 based on the molecular docking model between 5′tRF‐GlyGCC and LDHA. Subsequently, we utilized the RIP and biotin‐pull down assay to examine the binding capability of 5′tRF‐GlyGCC with LDHA‐Mut‐3. Notably, the binding affinity of 5′tRF‐GlyGCC to LDHA‐Mut‐3 was significantly diminished when compared to that with the full‐length LDHA (Figure 3G–I). The outcomes indicated that the 15th to 20th amino acids of LDHA are crucial for 5′tRF‐GlyGCC binding to LDHA.

Subsequently, we constructed seven mutants of 5′tRF‐GlyGCC (Figure 3J) and used the biotin RNA‐pull down assay to detect the key binding sites of LDHA on 5′tRF‐GlyGCC. The results indicated that the nucleotide positions 14, 15, and 16 of 5′tRF‐GlyGCC are crucial for binding to LDHA (Figure 3K). These results demonstrate that 5′tRF‐GlyGCC specifically binds to LDHA in a sequence‐dependent manner.

### 2.4 FGFR1 Mediates 5′tRF‐GlyGCC‐Induced LDHA Activation

**Figure 4:**
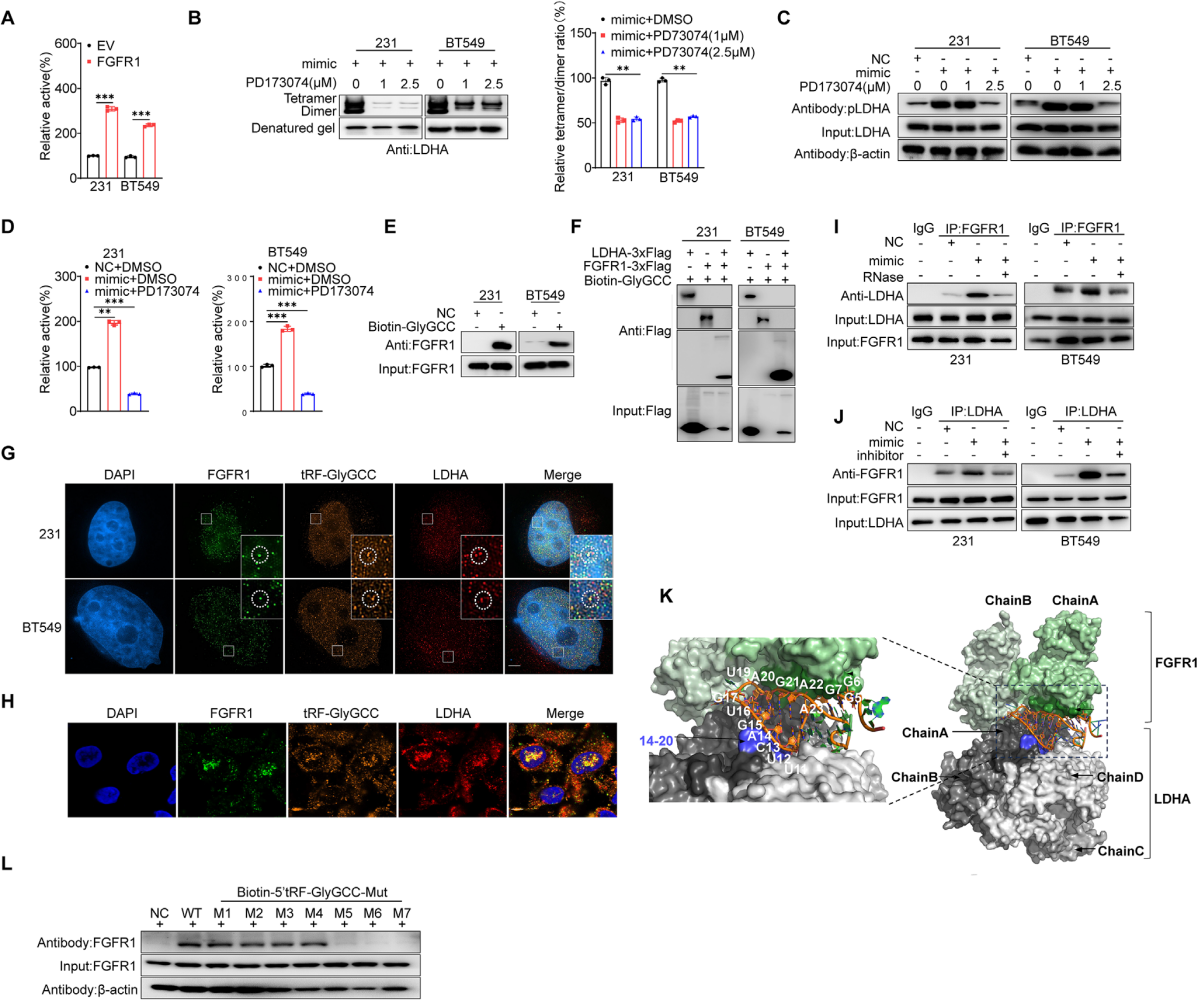
FGFR1 Mediates 5′tRF‐GlyGCC‐Induced LDHA Activation. A) Endogenous LDHA protein activity was evaluated in MDA‐MB‐231 and BT549 cells subsequent to FGFR1 overexpression. B) Native Western blot analysis was conducted to evaluate (left panels) and quantitatively determine (right panels) the changes in LDHA tetramers in MDA‐MB‐231 (left) and BT549 (right) cells following transfection with the 5′tRF‐GlyGCC mimic for 48 h, subsequent to which the cells were treated with the FGFR1 inhibitor PD173074 for 24 h. C) Western blot analysis of products from RNA‐pulldown assays using 5′tRF‐GlyGCC or a nontargeting oligo to assess changes in phosphorylation of LDHA protein in MDA‐MB‐231(left) and BT549 (right) cells after transfection with the 5′tRF‐GlyGCC mimic for 48 h, followed by treatment with the FGFR1 inhibitor PD173074 for 24 h. D) The activity of endogenous LDHA protein was analyzed in MDA‐MB‐231(left) and BT549 (right) cells after transfection with the 5′tRF‐GlyGCC mimic for 48 h, followed by treatment with the FGFR1 inhibitor PD173074 for 24 h. E) Western blot analysis was performed on products from RNA‐pulldown assays using either 5′tRF‐GlyGCC or a nontargeting oligo to assess the binding interaction between 5′tRF‐GlyGCC and FGFR1. F) A pull‐down assay was conducted using Flag‐tagged recombinant FGFR1 protein, Flag‐tagged recombinant LDHA, and biotin‐labeled 5′tRF‐GlyGCC within MDA‐MB‐231 (left) and BT549 (right) cells. G) The cellular localizations of 5′tRF‐GlyGCC, LDHA, and FGFR1 were analyzed by combining RNA‐FISH and immunofluorescence. Cell nuclei were stained with DAPI, and the scale bar was 10 µm. H) The localization of 5′tRF‐GlyGCC, LDHA, and FGFR1 in human BC clinical specimens was analyzed using a combination of RNA‐FISH and immunofluorescence. The scale bar represents 10 µm. I) MDA‐MB‐231 (left) and BT549 (right) cells were treated with RNase, and the interaction between LDHA and FGFR1 was detected by co‐IP assay using anti‐FGFR1 antibody. J) MDA‐MB‐231 (left) and BT549 (right) cells were transfected with 5′tRF‐GlyGCC inhibitor, and the interaction between LDHA and FGFR1 was detected by co‐IP assay using anti‐LDHA antibody. K) The predicted dominant binding mode of LDHA‐5′tRF‐GlyGCC ‐FGFR1 complex. The LDHA and FGFR1 proteins are shown on the surface with different colors for different chains, and 5′tRF‐GlyGCC is shown by orange ribbons. The key amino acid residues on LDHA and FGFR1 that interact with 5′tRF‐GlyGCC are shown by highlighted by blue and green surfaces, respectively. The nucleotide residues that form hydrogen bond and salt bridge interactions with LDHA and FGFR1 are shown by orange sticks. L) Western blot analysis was performed on products from RNA‐pulldown assays to examine the binding of various 5′tRF‐GlyGCC mutants to FGFR1 in MDA‐MB‐231 cells. Data are presented as mean ± SD from three independent experiments. ^*^ *p* < 0.05, ^**^ *p* < 0.01, ^***^ *p* < 0.001, ns, no significant.

It has been reported that the phosphorylation of LDHA at Tyr10 is primarily regulated by fibroblast growth factor receptor 1 (FGFR1), which promotes LDHA's enzymatic activity in the glycolysis process.^[^ ^8^ ^]^ Our results confirmed that the overexpression of FGFR1 in BC cells significantly enhanced the enzymatic activity of LDHA (**Figure** **4**A). Given that LDHA tetramerization is determined by FGFR1‐catalyzed phosphorylation of LDHA at Tyr10,^[^ ^30^ ^]^ we tested whether PD173074, an FGFR1 inhibitor, would affect LDHA's phosphorylation and tetramerization. The results showed that FGFR1 inhibition suppressed LDHA tetramerization (Figure 4B) and phosphorylation at Tyr10 (Figure 4C) in BC cells. Our data verified that FGFR1 can phosphorylate LDHA at Tyr10 to enhance its activity in BC cells.

We then further examined whether FGFR1 mediated 5′tRF‐GlyGCC‐induced activation of LDHA. The FGFR1 inhibitor significantly reversed the activation of LDHA by 5′tRF‐GlyGCC (Figure 4D). Further, in biotin‐pull‐down assays, 5′tRF‐GlyGCC directly bound FGFR1 in BC cells (Figure 4E). To further elucidate the binding pattern among 5′tRF‐GlyGCC, LDHA, and FGFR1, we employed Flag‐pull‐down experiments to assess their interaction, and observed direct binding of 5′tRF‐GlyGCC to both LDHA and FGFR1, suggesting the formation of a ternary protein complex by the three molecules (Figure 4F). Subsequently, RNA‐FISH combined with immunofluorescence assays revealed that 5′tRF‐GlyGCC, LDHA, and FGFR1 colocalized in the nucleus of BC cells (Figure 4G). To further validate these findings, we performed these experiments using clinical samples of BC patients and obtained consistent results showing the nuclear colocalization of these three molecules (Figure 4H).

To determine whether 5′tRF‐GlyGCC mediates the ternary complex formation, we performed immunoprecipitation assays following RNase treatment to assess the binding of LDHA and FGFR1. The data revealed that RNase significantly reduced the binding of LDHA and FGFR1 (Figure 4I). Concurrently, to verify whether the structural integrity of 5′tRF‐GlyGCC is essential for its binding to FGFR1/LDHA and for exerting its functional effects, we synthesized a tRF with a scrambled control sequence (SC). Only the 5′tRF‐GlyGCC inhibitor, but not the no‐target control (NC) or SC, significantly attenuated LDHA‐FGFR1 binding in BC cells (Figure S3A, Supporting Information). Subsequently, we assessed the impact of 5′tRF‐GlyGCC inhibitor on the binding of LDHA and FGFR1 using immunoprecipitation, and observed that the inhibitor significantly decreased their interaction (Figure 4J). To further evaluate the function of 5′tRF‐GlyGCC on the signaling between LDHA and FGFR1, we investigated the potential binding pose (Figure 4K) of FGFR1 on the LDHA‐5′tRF‐GlyGCC complex by docking FGFR1 to the LDHA‐5′tRF‐GlyGCC complex through the HDOCK procedure (Figure 3A). The statistical analysis revealed that nucleotides G5‐G7 and G18‐A22 on 5′tRF‐GlyGCC are the key residues that form the hydrogen bonds and salt bridges (Figure 4K). Subsequent analyseis demonstrated that 5′tRF‐GlyGCC functions as a scaffold that bridges FGFR1 and LDHA, and that 5′tRF‐GlyGCC is capable of disrupting the interaction between FGFR1 and LDHA.

To further evaluate the role of 5′tRF‐GlyGCC in the signaling crosstalk between LDHA and FGFR1, we investigated the potential binding conformation of FGFR1 on the LDHA‐5′tRF‐GlyGCC complex (Figure 3A) by performing molecular docking of FGFR1 to this complex using the HDOCK docking protocol. The predicted binding pose and statistical analysis revealed that nucleotides G5–G7 and G18–A22 of 5′tRF‐GlyGCC serve as key residues mediating its hydrogen bond and salt bridge interactions with FGFR1 (Figure 4K).

To validate these docking results, we conducted site‐directed mutagenesis by mutating the key nucleotides of 5′tRF‐GlyGCC implicated in its binding to LDHA. At the LDHA‐5′tRF‐GlyGCC interface, U12 of the WT 5′tRF‐GlyGCC sequence forms a hydrogen bond with Asn130 of LDHA (Figure S3B, Supporting Information). Substituting U12 with a cytosine (i.e., the U12C mutation) abrogated this hydrogen bond and significantly reduced the interfacial interaction between 5′tRF‐GlyGCC and LDHA. Additional mutations within this region, including C13U, A14G, G15A, U16C, and G17A (Figure S3C–G, Supporting Information), elicited comparable effects of the reduced interaction. Collectively, these data demonstrate the crucial role of the U12–G17 segment in mediating the binding of 5′tRF‐GlyGCC to LDHA.

At the FGFR1‐5′tRF‐GlyGCC interface, mutations at the positions G5–G7 and G18 of 5′tRF‐GlyGCC (Figure S3H–K, Supporting Information) disrupted multiple hydrogen bonds, thereby reducing the binding between 5′tRF‐GlyGCC and FGFR1.

Taken together, these mutagenesis analyses confirmed that the U12–G17 region of 5′tRF‐GlyGCC at the LDHA interface and the specific nucleotides (G5–G7, G18) residing at the FGFR1 interface are critical for 5′tRF‐GlyGCC to bind LDHA and FGFR1, respectively. These findings provide important mechanistic evidence to support the structural and functional underpinnings of the proposed RNA scaffolding model.

To further elucidate the binding site of FGFR1 on 5′tRF‐GlyGCC, we conducted a biotin‐pull‐down experiment using previously generated truncated mutants of 5′tRF‐GlyGCC (Figure 3J). The findings revealed that FGFR1 directly engages with nucleotides 17, 18, and 19 of 5′tRF‐GlyGCC (Figure 4L), which are broadly consonant with our molecular docking studies. Collectively, these results demonstrate that 5′tRF‐GlyGCC acts as a molecular scaffold to build the FGFR1‐LDHA bridge and promote LDHA phosphorylation at Tyr10, subsequently facilitating its tetramerization and enhancing its enzymatic activity.

### 2.5 5′tRF‐GlyGCC/LDHA Induces Infiltration and Polarization of Macrophages

**Figure 5:**
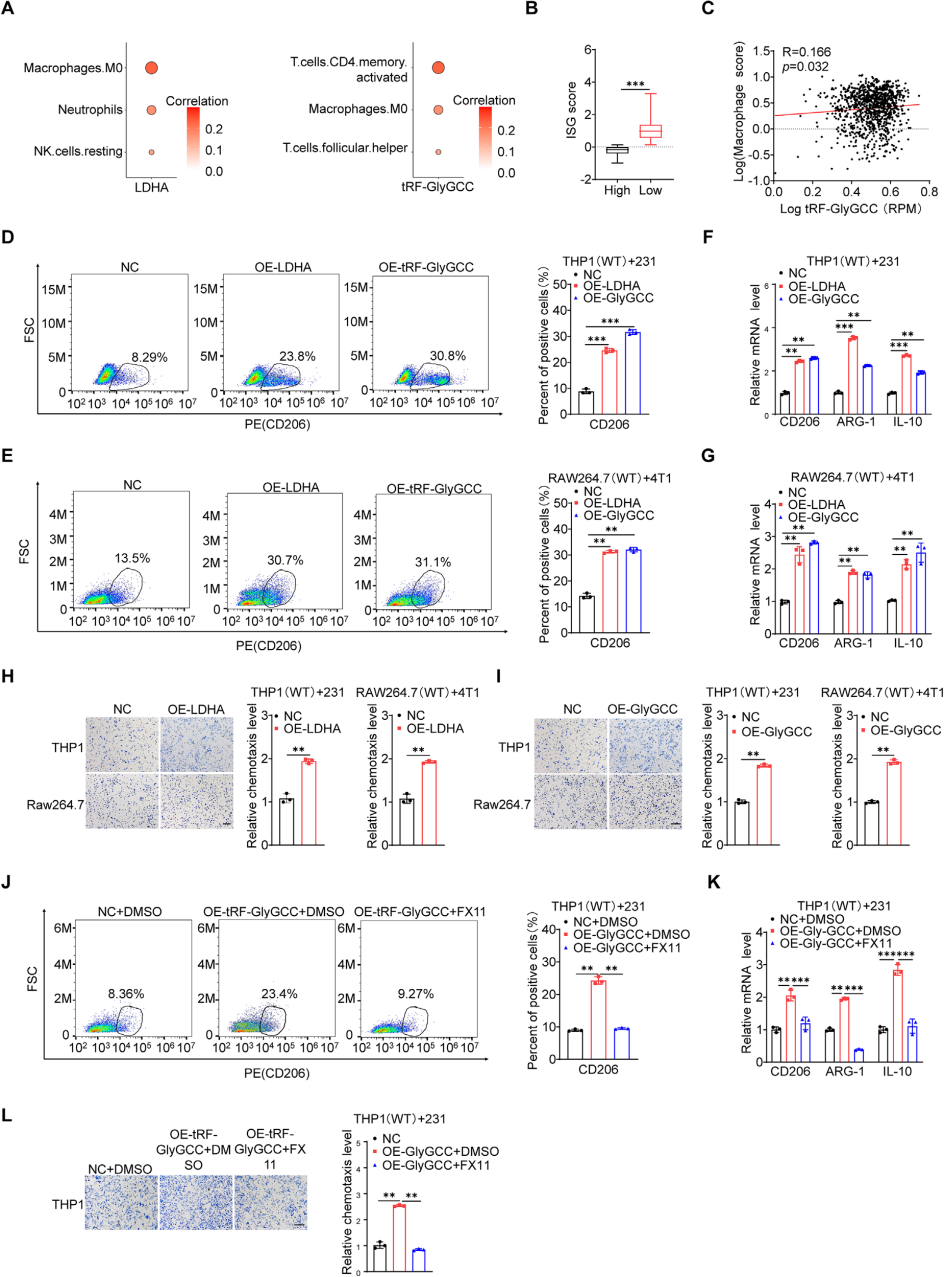
5′tRF‐GlyGCC/LDHA Induces Infiltration and Polarization of Macrophages. A) Heatmap showing immune cells with significant positive correlation with 5′tRF‐GlyGCC and LDHA gene expression. R>0.12, *p* values < 0.05. B) 5′tRF‐GlyGCC expression in the TCGA BRCA cohort negatively correlates with ISG score. According to the lower quartile of 5′tRF‐GlyGCC expression, patients were divided into high and low expression groups. 5′tRF‐GlyGCC high group: n = 898, 5′tRF‐GlyGCC low group: n = 300. Data are presented as box plots. The central line within the boxplot signifies the median value, which divides the data into two equal halves. The box itself encompasses the first and third quartiles, representing the interquartile range. *p*‐values are calculated based on a two‐tailed Wilcoxon test to assess statistical significance. C) Scatter plot showing the correlation between 5′tRF‐GlyGCC and macrophage marker genes (CCL2, CD163, CD68, IL10, LOX, PLOD2 and SIGLEC1). Correlation is defined by Spearman's correlation coefficient. The x‐axis represents the expression level of 5′tRF‐GlyGCC, and the y‐axis represents indicates the mean of log2 (TPM + 1) of macrophage marker genes(ref) which was used as the expression of macrophage markers. The calculation method of the signature score is shown in the methods. D, E) Upon stimulating THP1 and RAW cells with conditioned media (CM) derived from MDA‐MB‐231 (D) and 4T1 (E) cells stably overexpressing LDHA and 5′tRF‐GlyGCC for 48 h, the alteration in the expression of the M2 macrophage surface marker CD206 was detected using a flow cytometer (left) and quantitatively analyzed (right). F, G) Upon stimulating THP1 and RAW cells with CM derived from MDA‐MB‐231 (F) and 4T1 (G) cells stably overexpressing LDHA and 5′tRF‐GlyGCC for 48 h, the alteration in the expression of the M2 macrophage markers was quantified through RT‐qPCR analysis. H, I) Representative images (left) and quantification (right) of relative migration of THP1 and Raw macrophages from a transwell analysis following stimulation with CM from MDA‐MB‐231 and 4T1 cells expressing NC and overexpression of LDHA (H) or 5’tRF‐GlyGCC (I). Scale bar, 100 µm. J, K) Upon stimulating THP1 cells with CM derived from MDA‐MB‐231 cells stably overexpressing 5′tRF‐GlyGCC, subsequent treatment with or without FX11 for 48 h, the expression of the M2 macrophage surface marker CD206 was detected using a flow cytometer (left) and quantitatively analyzed (right) (J), and the expression of the M2 macrophage markers was quantified through RT‐qPCR analysis (K). L) Representative images (left) and quantification (right) of relative migration of THP1 macrophages from a transwell analysis following stimulation with CM from MDA‐MB‐231 cells expressing NC and overexpression of 5′tRF‐GlyGCC, subsequent treatment with or without FX11 for 48 h. Scale bar, 100 µm. Data are presented as mean ± SD from three independent experiments. ^*^ *p* < 0.05, ^**^ *p* < 0.01, ^***^ *p* < 0.001, ns, no significant.

LDHA is critical for the immune cell infiltration in the tumor microenvironment,^[^ ^32^ ^]^ and our data showed that 5′tRF‐GlyGCC expression significantly correlated with cytokine‐cytokine receptor interaction (Figure 1F). Thus, we evaluated the potential roles of 5′tRF‐GlyGCC/LDHA in the BC microenvironment. Subsequent analysis revealed a significant correlation between LDHA and various immune cells, including macrophages M0, neutrophils, resting NK cells, resting mast cells, monocytes, NK cells activated, and T cells CD8 (Figure S4A, Supporting Information). Conversely, 5′tRF‐GlyGCC exhibited notable associations with T cells CD4 memory activation, Macrophages M0, Follicular Helper T cells, and resting mast cells (Figure S4B, Supporting Information). To delve deeper into the potential functions of LDHA and 5′tRF‐GlyGCC within the tumor microenvironment of BC, we intersected immune cells positively correlated with both LDHA and 5′tRF‐GlyGCC, with a correlation threshold of R > 0.12 and a *p*‐value < 0.05. Notably, macrophages displayed the highest correlation with LDHA and 5′tRF‐GlyGCC (**Figure** **5**A). Concurrently, our findings suggest that an increased expression of 5′tRF‐GlyGCC in BC correlated with a diminished ISG score (Figure 5B). There was a marked positive association between macrophage markers and 5′tRF‐GlyGCC (Figure 5C). These findings suggest that LDHA and 5′tRF‐GlyGCC potentially mediate the infiltration of macrophages.

Subsequently, we employed culture media sourced from MDA‐MB‐231 and 4T1 cells, which had been engineered to overexpress both 5′tRF‐GlyGCC and LDHA, in a co‐culture with THP1 and RAW cells for a duration of 48 h. Thereafter, we employed flow cytometry to ascertain the influence of the overexpression of 5′tRF‐GlyGCC and LDHA on the surface molecules of macrophages, with gating strategies displayed in Figure S4C (Supporting Information). The findings revealed that the overexpression of 5′tRF‐GlyGCC and LDHA markedly enhanced the expression of M2‐type surface molecule CD206 on macrophages (Figure 5D,E), without significantly affecting the M1‐type surface molecule CD86 (Figure S4D, Supporting Information). Next, we analyzed the correlation between 5′tRF‐GlyGCC and CD206, a well‐recognized marker of the M2‐type macrophages, and observed a statistically significant positive correlation between 5′tRF‐GlyGCC and CD206 (Figure S4E, Supporting Information). Following this, we stimulated THP1 and RAW cells with the culture media derived from MDA‐MB‐231 and 4T1 cells overexpressing 5′tRF‐GlyGCC and LDHA, assessing the impact of the overexpression on the M2‐type markers of macrophages. The results demonstrated that the overexpression of 5′tRF‐GlyGCC and LDHA significantly facilitated the expression of M2‐type markers CD206, ARG‐1, and IL‐10 (Figure 5F,G). Building on these findings, THP1 and RAW cells were stimulated by the culture media from 5′tRF‐GlyGCC‐ and LDHA‐overexpressing MDA‐MB‐231 and 4T1 cells, revealing significantly enhanced macrophage migration (Figure 5H,I). These findings suggest that LDHA and 5′tRF‐GlyGCC in BC cells can induce the polarization and migration of macrophages.

To further examine whether the promotion of macrophage M2 polarization by the 5′tRF‐GlyGCC/LDHA axis was a direct consequence of LDHA activation and subsequent lactate production, we first directly measured the lactate concentration in the culture medium of cells stably overexpressing 5′tRF‐GlyGCC or LDHA. Following stable overexpression of 5′tRF‐GlyGCC or LDHA in BC cells, the lactate levels in the culture media showed statistically significant increases (Figure S4F, Supporting Information).

Next, we cultivated THP1 cells using the culture medium from MDA‐MB‐231 cells subjected to either 5′tRF‐GlyGCC knockdown or LDHA inhibitor FX11 treatment. The data indicated statistically significant decreases of both macrophage chemotactic capacity (Figure S4G, Supporting Information) and the expression levels of M2 macrophage markers, including CD206, ARG‐1, and IL‐10 (Figure S4H,I, Supporting Information). Notably, exogenous lactate supplementation markedly reversed these inhibitory effects; specifically, it restored macrophage chemotactic capacity to near‐normal levels and induced a significant increase in the expression of the aforementioned M2 polarization markers (Figure S4H,I, Supporting Information).

To investigate the suppression status of CD8⁺ T cells in response to the 5′tRF‐GlyGCC/LDHA axis, we conducted co‐culture experiments using a system consisting of CD8⁺ T cells, M0/M2 macrophages, and target cells (i.e., MDA‐MB‐231 cells). As shown in Figure S4J,K (Supporting Information), compared with the control group (with only M0 macrophages), the group with M0 macrophages co‐cultured in the medium from MDA‐MB‐231 cells overexpressing LDHA and 5′tRF‐GlyGCC exhibited a statistically significant reduction of CD8⁺ T cell cytotoxic activity against the target cells. These findings demonstrate that 5′tRF‐GlyGCC acts in concert with LDHA to promote the evasion of anti‐tumor immunity by BC cells, primarily through inducing macrophage polarization toward the M2 phenotype.

We then evaluate whether LDHA is involved in 5′tRF‐GlyGCC‐induced infiltration and polarization of macrophages. In MDA‐MB‐231 cells overexpressing 5′tRF‐GlyGCC, we incorporated the LDHA inhibitor FX11 and utilized the resultant media to stimulate THP1 cells. The results showed that the surface molecule CD206 (Figure 5J), as well as the M2 markers CD206, ARG‐1, and IL‐10 (Figure 5K), and the migratory capacity of macrophages (Figure 5L), induced by the overexpression of 5′tRF‐GlyGCC, were significantly diminished upon the addition of FX11. These findings suggest that the 5′tRF‐GlyGCC/LDHA axis within BC cells is precipitating the infiltration and polarization of macrophages.

### 2.6 CCL7 Mediates 5′tRF‐GlyGCC/LDHA‐Driven Macrophage Infiltration and Polarization

**Figure 6:**
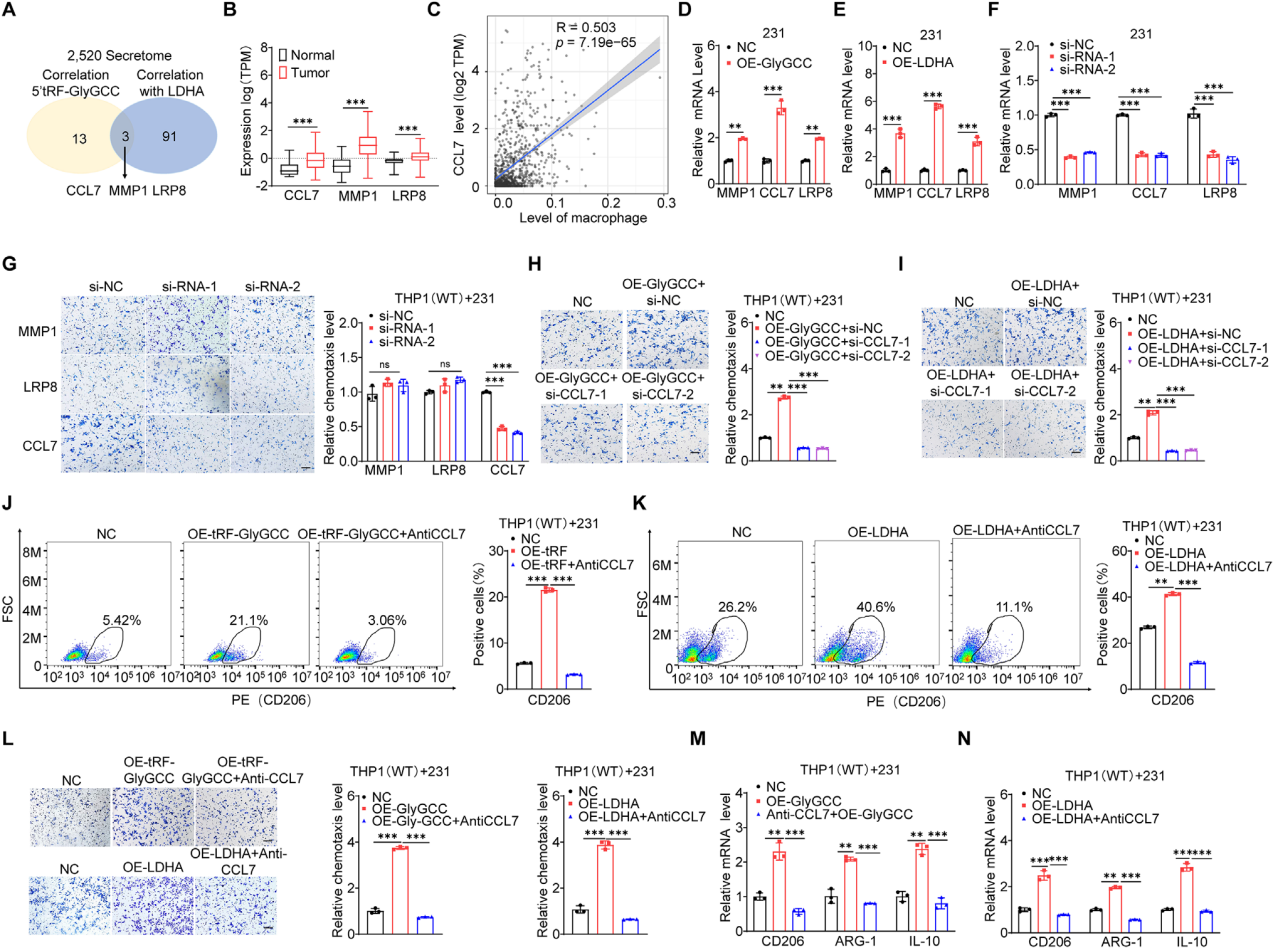
CCL7 Mediates 5′tRF‐GlyGCC/LDHA‐driven Macrophage Infiltration and Polarization. A) Venn diagram showing the process of selecting the chemokines and/or cytokines regulating macrophage recruitment in triple‐negative BC cells with high expression of LDHA and 5′tRF‐GlyGCC. The positive correlation between 5′tRF‐GlyGCC or LDHA expression and chemokines and/or cytokines was calculated by the Spearman correlation coefficient. R>0.3, *p* values < 0.05. B) Box plot showing the expression of CCL8, MMP1, and LRP8 genes in TCGA BRCA tumor (n = 339) and normal (n = 354) samples. Black refers to normal samples, and red refers to tumor samples. C) The scatter plot shows the correlation between CCL7 gene expression and macrophage infiltration levels. D, E) qPCR analysis was conducted to evaluate the mRNA levels of CCL7, MMP1, and LRP8 in MDA‐MB‐231cells stably overexpressing 5′tRF‐GlyGCC (D) or LDHA (E). F) qPCR analysis was conducted to detect the changes in the mRNA levels after the transfection of siRNAs targeting MMP1, CCL7, and LRP8 at a concentration of 50 nm in MDA – MB – 231 cells. The detection was carried out 48 h after the transfection. G) Representative images (left) and quantification (right) of the relative migration of THP1 macrophages from a transwell analysis following stimulation with CM from MDA‐MB‐231 cells transfected with si‐NC, si‐CCL7, si‐MMP1, and si‐LRP8 at a concentration of 50 nm for 48 h. Scale bar, 100 µm. H, I) Representative images (left) and quantification (right) illustrating the relative migration of THP1 macrophages from a transwell analysis, stimulated with CM from MDA‐MB‐231 cells stably overexpressing 5′tRF‐GlyGCC (H) and LDHA (I) ‐which were transfected with si‐NC and si‐CCL7 at a concentration of 50 nm for 48 h. Scale bar, 100 µm. J, K) Upon stimulating THP1 cells with conditioned media CM (with 2 µg mL^−1^ anti‐CCL7 neutralizing antibody) derived from MDA‐MB‐231 cells stably overexpressing 5′tRF‐GlyGCC (J) and LDHA (K) for 48 h, the alteration in the expression of the M2 macrophage surface marker CD206 was detected using a flow cytometer (left) and quantitatively analyzed (right). L) Representative images (left) and quantification (right) illustrating the relative migration of THP1 macrophages from a transwell analysis, stimulated with CM (with 2 µg mL^−1^ anti‐CCL7 neutralizing antibody) from MDA‐MB‐231 cells stably overexpressing 5′tRF‐GlyGCC and LDHA. Scale bar, 100 µm. M, N) Upon stimulating THP1 cells with media CM derived from MDA‐MB‐231 cells stably overexpressing 5′tRF‐GlyGCC (M) or LDHA (N), subsequent treatment with anti‐CCL7 neutralizing antibody (2 µg mL^−1^), the expression of the M2 macrophage markers was quantified through RT‐qPCR analysis. Data are presented as mean ± SD from three independent experiments. ^*^ *p* < 0.05, ^**^ *p* < 0.01, ^***^ *p* < 0.001, ns, no significant.

To clarify the chemokines and/or cytokines regulating macrophage recruitment in BC cells with high expression of LDHA and 5′tRF‐GlyGCC, we analyzed the correlation of LDHA and 5′tRF‐GlyGCC with secreted proteins in the human secreted protein dataset (Table S4, Supporting Information).^[^ ^33^ ^]^ Among the genes encoding these secreted proteins, 94 were significantly positively correlated with LDHA, and 16 were significantly positively correlated with 5′tRF‐GlyGCC. The intersection analysis of the two gene groups revealed that 3 genes (CCL7, MMP1, and LRP8) showed significantly positive correlation with both 5′tRF‐GlyGCC and LDHA (**Figure** **6**A). Subsequently, we used the TCGA‐BRCA to analyze the expression levels of these intersection genes, and the results showed that CCL7, MMP1, and LRP8 were significantly increased in tumor tissues (Figure 6B). These results suggest that CCL7, MMP1, and LRP8 are potentially involved in mediating 5′tRF‐GlyGCC/LDHA‐induced macrophage polarization.

Since our results validated that the 5′tRF‐GlyGCC/LDHA interaction regulates the polarization of macrophages M2, we analyzed the correlation of these proteins with macrophages using the Timer2 website, and the results showed that CCL7 (Figure 6C), MMP1 (Figure S5A, Supporting Information), and LRP8 (Figure S5B, Supporting Information) were all significantly positively correlated with macrophage infiltration, with CCL7 showing the strongest correlation. Overexpression of LDHA and 5′tRF‐GlyGCC could significantly increase the expression of CCL7, MMP1, and LRP8 in both MDA‐MB‐231 (Figure 6D,E) and 4T1 (Figure S5C,D, Supporting Information) cells. To further validate their roles in 5′tRF‐GlyGCC‐induced macrophage infiltration, we individually knocked down CCL7, LRP8, and MMP1 in MDA‐MB‐231 cells using siRNAs (Figure 6F). Their conditioned medium was co‐cultured with THP1 cells, and the results showed that only CCL7 knockdown significantly affected the chemotactic ability of THP1 cells compared to the control (Figure 6G). These results indicate that CCL7 is involved in mediating 5′tRF‐GlyGCC/LDHA‐induced macrophage polarization.

Subsequently, the conditioned medium of MDA‐MB‐231 cells with silenced CCL7 after overexpression of 5′tRF‐GlyGCC was co‐cultured with THP1. The results showed that silencing CCL7 could rescue 5′tRF‐GlyGCC‐induced migration of THP1 cells (Figure 6H). Similarly, silencing CCL7 also rescued LDHA‐induced migration of THP1 cells (Figure 6I). Following these experiments, we also incubated THP1 cells with conditioned media from MDA‐MB‐231 cells overexpressing 5′tRF‐GlyGCC and LDHA, and then treated these cells with CCL7‐neutralizing antibodies. The findings indicated that the augmented surface expression of the M2 macrophage marker CD206, attributed to 5′tRF‐GlyGCC (Figure 6J) or LDHA (Figure 6K) overexpression, was significantly diminished upon CCL7 neutralization. Additionally, the augmented migratory capacity of the macrophages induced by 5′tRF‐GlyGCC or LDHA overexpression was significantly curtailed upon the introduction of the CCL7‐neutralizing antibody (Figure 6L). Notably, the application of a CCL7‐neutralizing antibody markedly attenuated the increased mRNA levels of CD206, ARG‐1, and IL‐10 induced by the overexpression of 5′tRF‐GlyGCC (Figure 6M) or LDHA (Figure 6N). In summary, these results indicate that CCL7 is involved in 5′tRF‐GlyGCC/LDHA‐induced macrophage infiltration and polarization.

### 2.7 ALKBH3/ANG Are Regulatory Factors for 5′tRF‐GlyGCC Biogenesis in BC Cells

**Figure 7:**
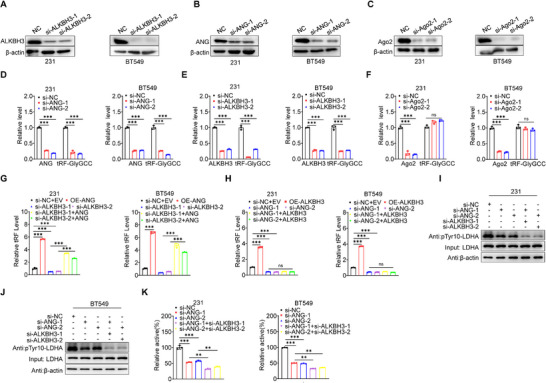
ALKBH3/ANG Are Regulatory Factors for 5′tRF‐GlyGCC Biogenesis in BC Cells. A–C) The protein expression of ALKBH3 (A), ANG (B), or AGO2 (C) in BC cells transfected with the negative control and 50 nm targeted siRNAs for 48 h. D–F) RT‐qPCR analysis of 5′tRF‐GlyGCC expression in BC cell line transfected with the negative control and targeted siRNAs for ANG (D), ALKBH3 (E), or AGO2 (F) at a concentration of 50 nm for 48 h. G, H) RT‐qPCR analysis of 5′tRF‐GlyGCC expression in BC cell line transfected with the negative control and targeted siRNAs at a concentration of 50 nm for 48 h and subsequent transfection with ANG (G) and ALKBH3 (H). I, J) Western blot analysis of LDHA phosphorylation in the MDA‐MB‐231 (I) and BT549 (J) cell lines, transfected with negative control siRNAs and targeted siRNAs at a concentration of 50 nm for 48 h. K) The activity of endogenous LDHA protein was analyzed in MDA‐MB‐231 (left) and BT549 (right) cells transfected with negative control siRNAs and targeted siRNAs at a concentration of 50 nm for 48 h. Data are presented as mean ± SD from three independent experiments. ^*^ *p* < 0.05, ^**^ *p* < 0.01, ^***^ *p* < 0.001, ns, no significant.

tRFs are a class of small RNAs produced when tRNA is enzymatically cleaved in response to stress. Increasing reports suggest that ALKBH3 can demethylase tRNA m^1^A to induce the cleavage of tRNA by ANG or Argonaute RISC catalytic component 2 (AGO2).^[^ ^34^ ^]^ Consistent with our previous findings, a significant correlation exists between the expression levels of ALKBH3 and the production of 5′tRF‐GlyGCC.^[^ ^35^ ^]^ Subsequently, our bioinformatics analysis of the TCGA datasets revealed that the expression levels of 5′tRF‐GlyGCC exhibited the most significant correlation with ANG in BC tissues (Table S5, Supporting Information). To further explore the mechanism of 5′tRF‐GlyGCC generation, we examined the effects of ALKBH3, AGO2, and ANG knockdown on 5′tRF‐GlyGCC expression in BC cells (**Figure** **7**A–C). Only the knockdown of ALKBH3 and ANG, but not AGO2, could significantly reduce the abundance of 5′tRF‐GlyGCC in BC cells (Figure 7D–F). When further investigating the regulatory mechanisms of ALKBH3 and ANG on 5′tRF‐GlyGCC, we discovered that complementing ANG in BC cells after knocking down ALKBH3 significantly increased the abundance of 5′tRF‐GlyGCC (Figure 7G), whereas complementing ALKBH3 in BC cells with ANG knockdown had no effect on 5′tRF‐GlyGCC levels (Figure 7H). These findings suggest that 5′tRF‐GlyGCC is potentially generated through ALKBH3‐mediated tRNA demethylation followed by ANG‐dependent cleavage.

We further checked whether ALKBH3 and ANG could modulate the expression and activity of LDHA. The knockdown of ALKBH3 and ANG in BC cells significantly reduced LDHA phosphorylation (Figure 7I,J) and activity (Figure 7K). When both ALKBH3 and ANG were knocked down concurrently in BC cell lines, we detected a more pronounced and significant decrease of LDHA phosphorylation (Figure 7I,J) and activity (Figure 7K) compared to the situation with ALKBH3 or ANG individually silenced. These results suggest that ALKBH3/ANG are regulatory factors for 5′tRF‐GlyGCC biogenesis and LDHA activity in BC cells.

### 2.8 Targeted Suppress BC Progression Based on 5′tRF‐GlyGCC/LDHA Signals

**Figure 8:**
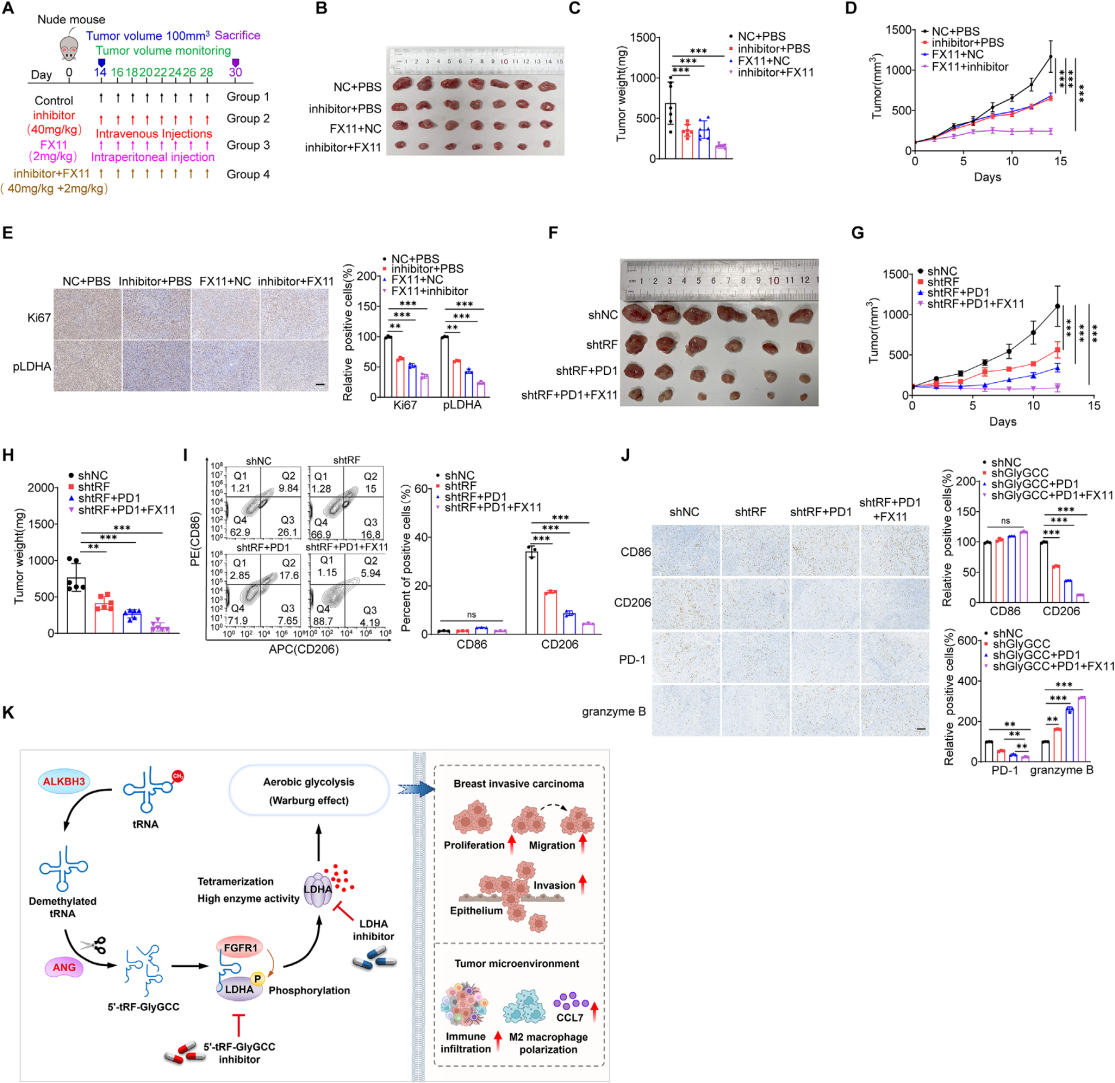
Targeted Suppress BC Progression Based on 5′tRF‐GlyGCC/LDHA Signals. A) Schematic representation of the treatment regimen for mice harboring xenografts derived from MDA‐MB‐231 cells, which were intravenously administered with the 5′tRF‐GlyGCC inhibitor or a control agent. The colored arrows denote the timing of dosing and the monitoring intervals for tumor measurements. B–D) Representative images of mice bearing tumors (B), tumor weight (C), and tumor volume (D) of mice inoculated with MDA‐MB‐231 cells. E) IHC was employed to assess(left) and quantitatively analyze (right) the alterations in the phosphorylation of LDHA and Ki67 within the tumor tissue. Scale bars: 100 µm. F–H) Representative images of mice bearing tumors (F), tumor volume (G), and tumor weight (H) of mice inoculated with 4T1‐shNC, 4T1‐sh5’tRF‐GlyGCC, 4T1‐sh5’tRF‐GlyGCC+PD1, and 4T1‐sh5’tRF‐GlyGCC+PD1+FX11 cells. I) The Percentage of CD8^+^, CD206^+^, and F4/80^+^ macrophages in CD45^+^ immune cells was analyzed by flow cytometry. Tumors were harvested on day 19 after the tumor cells. CD86‐PE, F4/80‐Percp/cy5.5, CD206‐APC, and CD45‐FITC were used to mark macrophages in the tumor (left) and quantitatively analyzed (right). J) Immunohistochemistry was employed to assess(left) and quantitatively analyze (right) the alterations in the CD86, CD206, granzyme B, and PD‐1within the tumor tissue. Scale bars:100 µm. K) A proposed mechanism model illustrating the impact of increased 5′tRF‐GlyGCC on the progression of BC. Data are presented as mean ± SD from three independent experiments. ^*^ *p* < 0.05, ^**^ *p* < 0.01, ^***^ *p* < 0.001, ns, no significant.

Given the strong promoting effect of 5′tRF‐GlyGCC on BC, we established an MDA‐MB‐231 cell xenograft model and treated mice with a 5′tRF‐GlyGCC inhibitor. We further explored the effects of combined treatment of FX11 and 5′tRF‐GlyGCC inhibitors on the growth of subcutaneous xenografts in nude mice (**Figure** **8**A). The treatment was started when the tumor volume reached ≈100 mm^3^. Single use of FX11 (2 mg kg^−1^) or 5′tRF‐GlyGCC (40 mg kg^−1^) inhibitors significantly inhibited the growth of subcutaneous xenografts formed by the MDA‐MB‐231 cells, with a significant reduction in tumor size (Figure 8B), weight (Figure 8C), and volume (Figure 8D). Compared with single inhibitor treatment, the combined treatment of FX11 and 5′tRF‐GlyGCC inhibitors had a more significant inhibitory effect on the growth of subcutaneous BC xenografts (Figure 8B–D), and there was no significant effect on mouse weight (Figure S6A, Supporting Information). IHC showed that single use of FX11 or 5′tRF‐GlyGCC inhibitors significantly reduced the phosphorylation level of LDHA and Ki67. Compared with the single inhibitor treatment, the combination of FX11 and 5′tRF‐GlyGCC inhibitors exhibited a greater effect to inhibit the phosphorylation of LDHA and expression of Ki67 (Figure 8E).

To further explore the effects of the 5′tRF‐GlyGCC/LDHA axis on BC progression, we performed subcutaneous tumor experiments in Balb/c mice, injecting stable knockdown 5′tRF‐GlyGCC and control group 4T1 cells into the fat pads of Balb/c mice and dividing the mice into four groups (shNC, sh5’tRF‐GlyGCC, sh5’tRF‐GlyGCC +PD1, and sh5’tRF‐GlyGCC +PD1+FX11). The results demonstrated that the tumor size (Figure 8F), volume (Figure 8G), and weight (Figure 8H) in the sh5’tRF‐GlyGCC group were significantly reduced compared to the shNC group. Furthermore, when compared to the sh5’tRF‐GlyGCC group, the sh5’tRF‐GlyGCC+PD1 and sh5’tRF‐GlyGCC+PD1+FX11 groups exhibited a further significant decrease in tumor size (Figure 8F), volume (Figure 8G), and weight (Figure 8H), with no significant impact on the body weight (Figure S6B, Supporting Information) of the mice. The data demonstrate that targeting 5′tRF‐GlyGCC/LDHA signals can suppress the growth of BC xenograft and enhance immunotherapy sensitivity.

To further evaluate the effects of the 5′tRF‐GlyGCC/LDHA axis on the tumor microenvironment, on the 19th day after combined treatment with FX11 and PD1, we performed flow cytometry to detect the proportions of CD45, CD8, CD206, and F4/80 cells, with gating strategies displayed in Figure S6C (Supporting Information). The results revealed that compared to the shNC group, the surface molecule CD206 of M2 macrophages in the sh5’tRF‐GlyGCC group was significantly decreased, while the surface molecule CD86 of M1 macrophages showed no significant change (Figure 8I). Furthermore, when compared to the sh5’tRF‐GlyGCC group, the sh5’tRF‐GlyGCC+PD1 and sh5’tRF‐GlyGCC+PD1+FX11 groups exhibited a further reduction in the surface molecule CD206 of M2 macrophages, with no significant effect on the surface molecule CD86 of M1 macrophages (Figure 8I). IHC analysis indicated that, when compared to the shNC and sh5’tRF‐GlyGCC groups, the sh5’tRF‐GlyGCC+PD1 and sh5’tRF‐GlyGCC+PD1+FX11 groups demonstrated a further reduction in the surface molecule CD206 of M2 macrophages, with no significant effect on the surface molecule CD86 of M1 macrophages (Figure 8J). Additionally, the expression of granzyme B was significantly upregulated, an effect attributed to the activation of cytotoxic immune cells; concomitantly, the expression of PD‐1 was downregulated (Figure 8J). These results indicate that the inhibition of 5′tRF‐GlyGCC in combination with anti‐PD‐1 and LDHA inhibitor FX11 can exert a synergistic effect in 4T1 subcutaneous tumors and reduce the infiltration of M2 macrophages.

## 3 Discussion

In the present study, we first analyzed the differences in basal 5′tRF‐GlyGCC expression across distinct BC subtypes using clinical samples from publicly available databases, and subsequently examined the functional effects of these differences using various experimental approaches. Based on these data, we propose the hypothesis that the 5′tRF‐GlyGCC/LDHA regulatory axis plays a functionally critical role in the BC subtypes with high glycolytic dependency, such as TNBC. Concurrently, our study uncovered a novel tumor‐promoting function of 5′tRF‐GlyGCC in BC progression, which is mediated by its interaction with the glycolytic enzyme LDHA and the subsequent reprogramming of the tumor microenvironment.

Previous studies have revealed that the majority of functionally characterized tRFs typically exert their activities through regulating mRNA stability, translation, or epigenetic processes via base‐pairing mechanisms, which resemble the actions of microRNAs. The regulatory processes include binding to mRNA 3′‐UTRs to suppress translation,^[^ ^20^, ^36^, ^37^ ^]^ displacing RNA‐binding proteins (such as YBX1) to destabilize oncogenic transcripts,^[^ ^25^ ^]^ and disrupting mitochondrial tRNAs and leucyl‐tRNA synthetase 2 (LaRS2) interactions to block mitochondrial translation.^[^ ^38^ ^]^ In contrast, our study uncovers a distinct functional paradigm in which 5′tRF‐GlyGCC acts as a protein‐complex scaffold, representing a unique and previously unappreciated mode of action for tRFs.

Specifically, we demonstrate that 5′tRF‐GlyGCC serves as a protein‐interaction scaffold that facilitates FGFR1‐mediated phosphorylation of LDHA at Tyr10, which enhances LDHA's enzymatic activity and promotes its tetramerization, key steps to promote glycolytic flux in tumor cells. This finding constitutes a significant advancement in elucidating the non‐canonical roles of tRFs, as it expands their functional repertoire beyond the RNA‐centric regulation (e.g., mRNA targeting or translation control) to direct modulation of protein‐protein interactions and post‐translational modifications.

The regulatory role of LDHA in aerobic glycolysis and the Warburg effect is well‐documented, with phosphorylation at Tyr10 by FGFR1 known to enhance its activity and cancer cell metastasis.^[^ ^27^, ^39^ ^]^ However, the upstream mechanisms governing FGFR1‐LDHA interaction remain poorly understood. Our discovery that 5′tRF‐GlyGCC acts as a molecular scaffold bridging FGFR1 and LDHA provides critical mechanistic insight into this process. Unlike previously reported kinase‐substrate interactions that solely rely on protein domains or adaptors, our work identifies a tRNA‐derived RNA as a novel facilitator of oncogenic signaling. This finding complements recent studies highlighting metabolic enzyme regulation by non‐coding RNAs^[^ ^14^, ^40^, ^41^, ^42^ ^]^ but uniquely positions 5′tRF‐GlyGCC as a dual modulator of both enzymatic activity (via phosphorylation) and structural organization (via tetramerization) of LDHA.

Notably, our study discovered a critical link between cancer metabolism and tumor immune evasion. While it is well‐established that lactate production driven by LDHA promotes the polarization of TAMs toward an immunosuppressive M2 phenotype,^[^ ^14^, ^43^ ^]^ we further identified a novel and direct connection between 5′tRF‐GlyGCC/LDHA signaling and chemokine‐mediated macrophage recruitment, specifically through upregulating the chemokine CCL7.

Besides macrophage‐centric immunosuppression, the programmed cell death protein 1 (PD‐1)/programmed cell death‐ligand 1 (PD‐L1) pathway represents another core mechanism underlying tumor immune evasion: PD‐1, a receptor expressed on the surface of activated T cells, interacts with its ligand PD‐L1 to suppress T cell effector function.^[^ ^44^, ^45^ ^]^ Notably, elevated PD‐1 expression on activated T cells further exacerbates this immune evasion phenotype.

In the present work, we show that the 5′tRF‐GlyGCC/LDHA/CCL7 signaling axis directly promotes M2 polarization of macrophages, thereby impairing CD8^+^ T cell‐mediated tumor cytotoxicity and reinforcing tumor immune evasion. This finding broadens the current paradigm of metabolic‐immune crosstalk by uncovering a tRF‐dependent regulatory mechanism that orchestrates glycolytic reprogramming (via LDHA) and immune microenvironment remodeling (via M2 macrophage polarization) in a coordinated manner.

Notably, our in vivo data demonstrate that dual inhibition of 5′tRF‐GlyGCC and LDHA jointly reduces both tumor growth and M2 macrophage polarization. This observation underscores the therapeutic potential of targeting the 5′tRF‐GlyGCC/LDHA/CCL7 axis to overcome immunotherapy resistance, a major clinical challenge in BC treatment.^[^ ^14^, ^46^ ^]^

The identification of ALKBH3/ANG as regulators of 5′tRF‐GlyGCC biogenesis further connects RNA modification machinery to cancer metabolism. While ALKBH3 is recognized for its role in tRNA demethylation and translation regulation,^[^ ^47^ ^]^ its involvement in generating oncogenic tRFs adds a new layer to its functional complexity. This combination of the identification aligns with emerging evidence that dysregulation of tRNA‐processing enzymes contributes to cancer progression^[^ ^47^, ^48^, ^49^ ^]^ but distinguishes our work by linking epitranscriptomic control to metabolic kinase signaling.

In summary, we have identified a tRF, 5′tRF‐GlyGCC, which plays a significant role in the development and progression of BC, and elucidated the molecular mechanisms by which 5′tRF‐GlyGCC facilitates the growth and metastasis of BC. Notably, significant therapeutic effects were observed upon treating xenografted BC cells in mice with an inhibitor of 5′tRF‐GlyGCC synthesized in vitro, thereby further validating the role of this tRF in BC and suggesting its potential as a therapeutic agent.

## 4 Experimental Section

### Transient Transfection of 5′tRF‐GlyGCC Mimic, Inhibitor, and Plasmids

5′tRF‐GlyGCC mimic and inhibitor were synthesized by GenePharma. Subsequently, cDNAs encoding LDHA were cloned individually into the pcDNA3.1‐3×FLAG vector, resulting in the generation of pcDNA3.1‐3×FLAG‐LDHA vectors. A corresponding empty pcDNA3.1‐3×FLAG vector served as a control. For the transient transfection experiments, mimic (50 nm) and plasmids (1 µg µL^−1^) were utilized with Lipo2000 reagent. The NC sequences are as follows: 5′‐ACA CAT CAA ACT CCG ACC CCC TTT G AG GAG G‐3′; The mimic sequences are as follows: 5′‐GCA TGG GTG GTT CAG TGG TAG AAT TCT CGC C‐3′; The inhibitor sequences are as follows: 5′‐GGC GAG AAT TCT ACC ACT GAA CCA CCC ATG C‐3′.

### RNA Pulldown Assays

A Pierce Magnetic RNA‐Protein Pull‐Down Kit (Thermo Fisher, 20164) was employed for RNA precipitation assays. Biotinylated non‐targeting oligonucleotides and 5′tRF‐GlyGCC sequences (2 µg) were incubated with protein extracts derived from BC cells. Following the addition of streptavidin beads, the associated total proteins were subjected to mass spectrometry or Western blot analysis for 5′tRF‐GlyGCC. Using an analogous approach, biotinylated 5′tRF‐GlyGCC was incubated with protein extracts from cells transfected with full‐length, truncated, or mutated LDHA, and the associated total proteins were analyzed by Western blot.

### Molecular Interaction Analysis

Surface plasmon resonance (SPR) binding assays were conducted using the BiacoreTM T200 system. Initially, the in vitro synthesized biotin‐labeled 5′tRF‐GlyGCC mimic was immobilized on a streptavidin Biacore chip at a concentration of 800 nm. Subsequently, recombinant LDHA protein was diluted in PBS to various concentrations and maintained at a flow rate of 30 µL min^−1^ throughout the kinetic experiment. The contact time was set at 120 s, with a dissociation time of 200 s. The collected data were analyzed using Biacore T200 Evaluation Software 2.0 (GE Healthcare) and GraphPad Prism 7.0.

### RNA Fish and Immunofluorescence

The Ribo Fluorescent In Situ Hybridization Kit (RiboBio) was employed for FISH analysis. In essence, BC cells or tumor tissues were prepared by fixation and permeabilization, followed by an overnight hybridization with 5′tRF‐GlyGCC probes at 37 °C in a dark, humid environment. Subsequently, the nuclei were labeled with DAPI. For immunofluorescence, primary antibodies against LDHA and secondary antibodies (Invitrogen, A‐21206, 1:500) were utilized. The fluorescence signals were visualized via FITC and Cy3 channels, with further nuclear counterstaining using DAPI. The final images were captured using an LSM880 confocal microscope (Zeiss).

### Mouse Tumor Models and Treatment

BALB/c nude mice and BALB/c mice (4 weeks old) were purchased from Sun Yat‐sen University (Guangzhou, China) Animal Center and raised under pathogen‐free conditions. All animal experiments complied with Zhongshan School of Medicine Policy on Care and Use of Laboratory Animals. The experimental protocols were approved by the Institutional Animal Care and Use Committee (Nos. SYSU‐IACUC‐2024‐002885, SYSU‐IACUC‐2025‐000086, SYSU‐IACUC‐2025‐000532). Four distinct xenograft models of BC were established. For the subcutaneous xenograft model in BALB/c nude mice, 0.1 mL of cell suspension containing 2×10^6^ stable overexpressing 5′tRF‐GlyGCC MDA‐MB‐231 and control MDA‐MB‐231 cells was injected into the subcutaneous tissue of the back legs. Tumor growth was monitored once every three days from palpable tumor development, with tumor volume calculated using the formula V = 1/2×larger diameter × (smaller diameter).^2^ In a separate BALB/c mouse subcutaneous xenograft model (divided into four groups), 0.1 mL of cell suspension containing 2×10^6^ stable knockdown 5′tRF‐GlyGCC 4T1(randomly allocated into three groups) and control 4T1 (one group) cells were injected subcutaneously into the back legs. Treatment with PD1 and FX11 was initiated upon tumor volume reaching 100 mm^3^ to assess tumor growth. Tumors were harvested on day 21 post‐injection, and analysis was conducted using flow cytometry, hematoxylin and eosin (H&E) staining, and immunohistochemistry (IHC). For the lung metastasis model, 0.1 mL of cell suspension containing 1×10^6^ stable overexpressing 5′tRF‐GlyGCC highly metastatic MDA‐MB‐231 and control highly metastatic MDA‐MB‐231 cells were injected via the tail vein into female athymic nude mice. Lung metastasis was assessed on day 21 post‐injection by H&E staining. In the treatment model using an in vitro chemically synthesized 5′tRF‐GlyGCC inhibitor, 0.1 mL of cell suspension containing 2×10^6^ MDA‐MB‐231 cells was injected subcutaneously into immunodeficient mice. Once tumors reached 100 mm^3^, mice were randomized into four groups and treated with 5′tRF‐GlyGCC inhibitor control, 5′tRF‐GlyGCC inhibitor, FX11, or a combination of both. Tumors were harvested on day 14 post‐treatment for IHC analysis.

The synthetic single‐stranded 5′tRF‐GlyGCC inhibitor features two phosphorus‐sulfur bonds at its 5′ end, four phosphorus‐sulfur bonds and one cholesterol moiety at its 3′ end, and 2′‐O‐methyl modifications throughout its nucleotide sequence. The dosage was determined based on previous studies and preliminary animal experiments. The 5′tRF‐GlyGCC inhibitor and its control (40 mg kg^−1^, i.v.), dissolved in normal saline, were administered at 100 µL per mouse every two days, for a total of seven doses. FX11 (3 mg kg^−1^, i.p.) was dissolved in normal saline and administered in the same manner, also totaling seven doses. Anti‐PD‐1 treatment (10 mg kg^−1^, i.p.) was given every other day, amounting to seven doses in total. The anti‐mouse PD1(CD279) ‐InVivo and FX11 were purchased from Selleck.

### Sample Collection

Tumor tissue samples were collected from newly diagnosed breast cancer (BC) patients at Sun Yat‐sen University Cancer Center. All patients were diagnosed based on histopathological examination of biopsy specimens, and the tissue samples were obtained at the time of initial diagnosis, prior to any surgical intervention or radiochemotherapy. This study was conducted in accordance with the Declaration of Helsinki. Written informed consent was obtained from all participants, and the study protocol was approved by the Ethics Committee of Sun Yat‐sen University Cancer Center (G2024‐021‐01).

### Statistical Analyses

Data were presented as the mean ± standard deviation (SD) derived from a minimum of three independent trials, unless otherwise noted. Statistical assessments were conducted using a two‐tailed unpaired Student's t‐test for comparisons between two groups and one‐way ANOVA followed by Bonferroni correction for comparisons among multiple groups. Although the assumption of normality was made for data distribution, this was not formally verified. GraphPad software was utilized for all statistical analyses, with a *p*‐value of less than 0.05 considered indicative of statistical significance.

## Conflict of Interest

The authors declare no conflict of interest.

## Author Contributions

C.Y. and Y.L. contributed equally to this work. H.W., Z.C., L.G., and C.Y. designed and initiated the study. C.Y., Z.C., L.G., Y.L., Y.W., Y.T., J.Z., G.X., Z.W., X.C., L.W., Y.Z., F.T., and X.Y. performed experiments. C.Y., Z.C., L.T., J.Z., and J.L. wrote the paper. X.C. provided assistance in bioinformatics analysis. C.Y., Z.C., L.G., Y.L., G.X., and J.Z. are in charge of supervision as well as data curation.

## Supporting information



Supporting Information

Supplemental Table 1–5

## Data Availability

The data that support the findings of this study are available in the supplementary material of this article.
