# Supplementary material for: 5′tRF‐GlyGCC Promotes Breast Cancer Progression via LDHA‐Mediated Glycolysis and Macrophage Polarization
Source: Adv Sci (Weinh). 2025 Nov 27;13(9):e14031. doi: 10.1002/advs.202514031 (PMC12903995; doi:10.1002/advs.202514031)
Supplement: Supplementary file 1 — Supporting Information [file ADVS-13-e14031-s001.docx]

**Supplementary data for**

**5’tRF-GlyGCC Promotes Breast Cancer Progression via LDHA-Mediated Glycolysis and Macrophage Polarization**

**Yi et al**

**Figure S1**


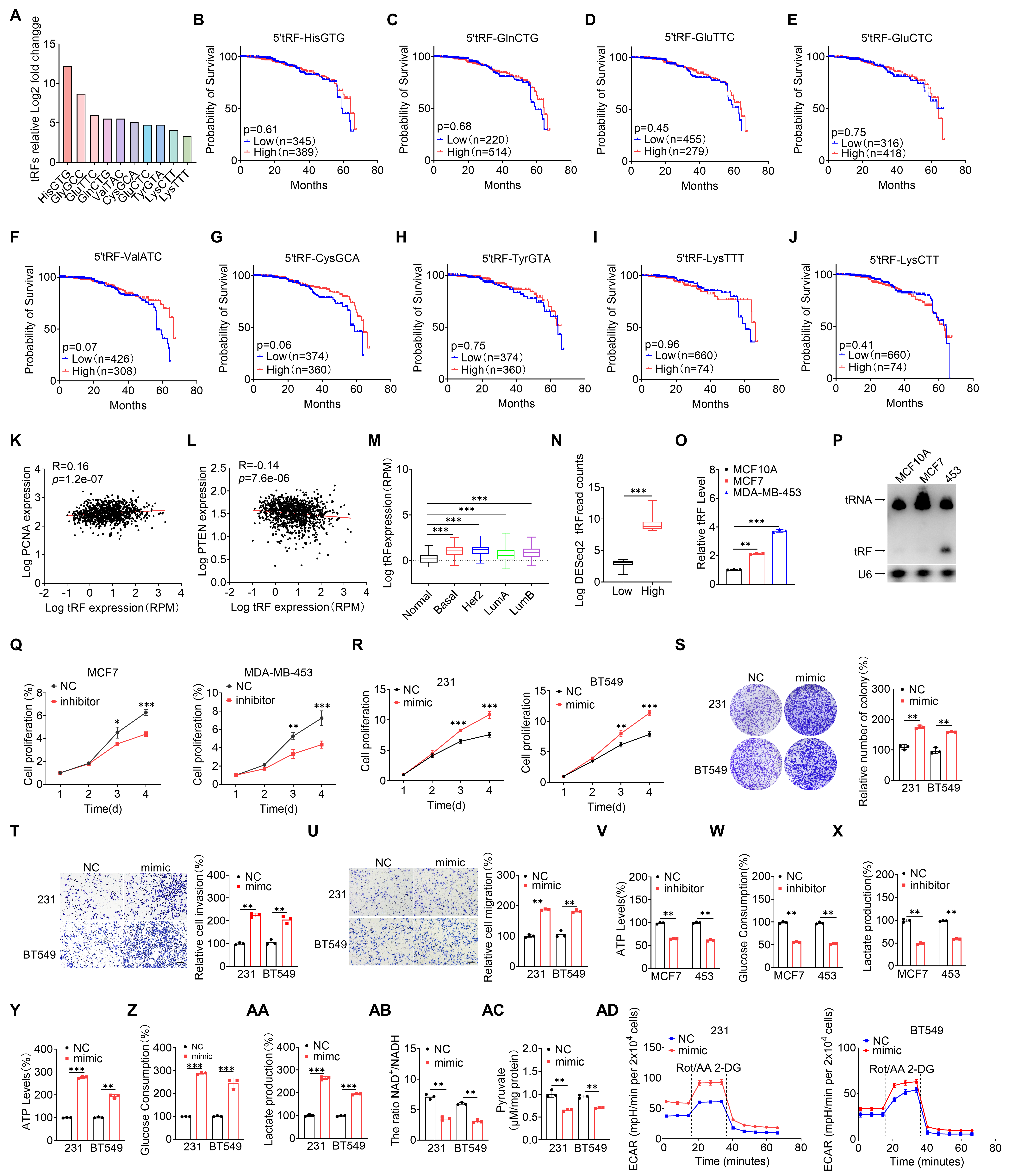


**Figure S1. 5’tRF-GlyGCC Promotes BC Progression by Enhancing Malignant Phenotypes and Metabolic Reprogramming**

A. The most highly expressed tRFs in the TCGA breast cancer database, ranked within the top ten, demonstrate substantial fold changes in their differential expression profiles.

B-J. Association of top ten tRFs expression with overall survival of patients in the TCGA-BRCA cohort. A log rank *P* < 0.05 was considered statistically significant.

K-L. Scatter plot analysis demonstrating the relationship between 5'tRF-GlyGCC expression levels and those of PCNA (K) and PTEN (L). Correlation is defined by spearman's correlation coefficient. R > 0 indicates positive correlation and R < 0 indicates negative correlation. *P* values < 0.05 were considered significant.

M. Expression of 5’tRF 5’tRF-GlyGCC in different subtypes of the TCGA BRCA cohort. LumA: Luminal A, n = 424; Lum B: Luminal B, n = 187; Basal: triple negative, n = 139; HER2+ non-luminal, n = 66; Normal, n = 90.

N. Box plot showing the relative abundance of 5’tRF-GlyGCC in the 10% 5’tRF-GlyGCC low and 5’tRF-GlyGCC high patients from the TCGA BRCA cohort. 94 patients with the highest and lowest 5’tRF-GlyGCC expression were assigned to each group. *P* values are determined by two-tailed Wilcoxon test. *** *P* < 0.001.

O. Levels of 5’tRF-GlyGCC in the MCF-10A, MCF7 and MDA-MB-453 cell lines by RT-qPCR analysis.

P. Levels of 5’tRF-GlyGCC in the MCF-10A, MCF7 and MDA-MB-453 cell lines by Northern blot analysis.

Q. CCK-8 assay was used to assess the proliferation of MCF7 and MDA-MB-453 cell lines following transfection with the 5’tRF-GlyGCC inhibitor.

R. The CCK-8 (Cell Counting Kit-8) assay was used to assess the proliferation of BC cell lines following transfection with the 5’tRF-GlyGCC mimic.

S. The clone formation assays (*left*) and their quantitative analysis (*right*) were utilized to evaluate the clonogenic potential of BC cells 48 hours after transfection with the 5’tRF-GlyGCC mimic.

T-U. The Transwell assays (*left*) and their quantitative analysis (*right*) were conducted to assess changes in the invasive (T) and migratory (U) abilities of BC cells 48 hours after transfection with the 5’tRF-GlyGCC mimic, scale bar: 100 μm.

V-X. Assays to detect ATP levels (V), glucose consumption (W), and lactate production (X) were performed to evaluate the changes in the metabolic profile of MCF7 and MDA-MB-453 cells 48 h following transfection with the 5’tRF-GlyGCC inhibitor.

Y-AA. The ATP levels (Y), glucose consumption (Z), and lactate production (AA) assays were performed to evaluate changes in the metabolic profile of BC cells 48 hours following transfection with the 5’tRF-GlyGCC mimic.

AB. The NAD⁺/NADH ratio assay was performed on MDA-MB-231 and BT549 cell lines subsequent to their transfection with 5’tRF-GlyGCC mimic.

AC. The pyruvate concentration assay was performed on MDA-MB-231 and BT549 cell lines subsequent to their transfection with 5’tRF-GlyGCC mimic.

AD. The ECAR assays were used to detect changes in glycolysis of BC cells following transfection with the 5’tRF-GlyGCC mimic.

Data are presented as mean ± SD from three independent experiments. **P*<0.05, ***P*<0.01, ****P*<0.001, ns, no significant.

**Figure S2**


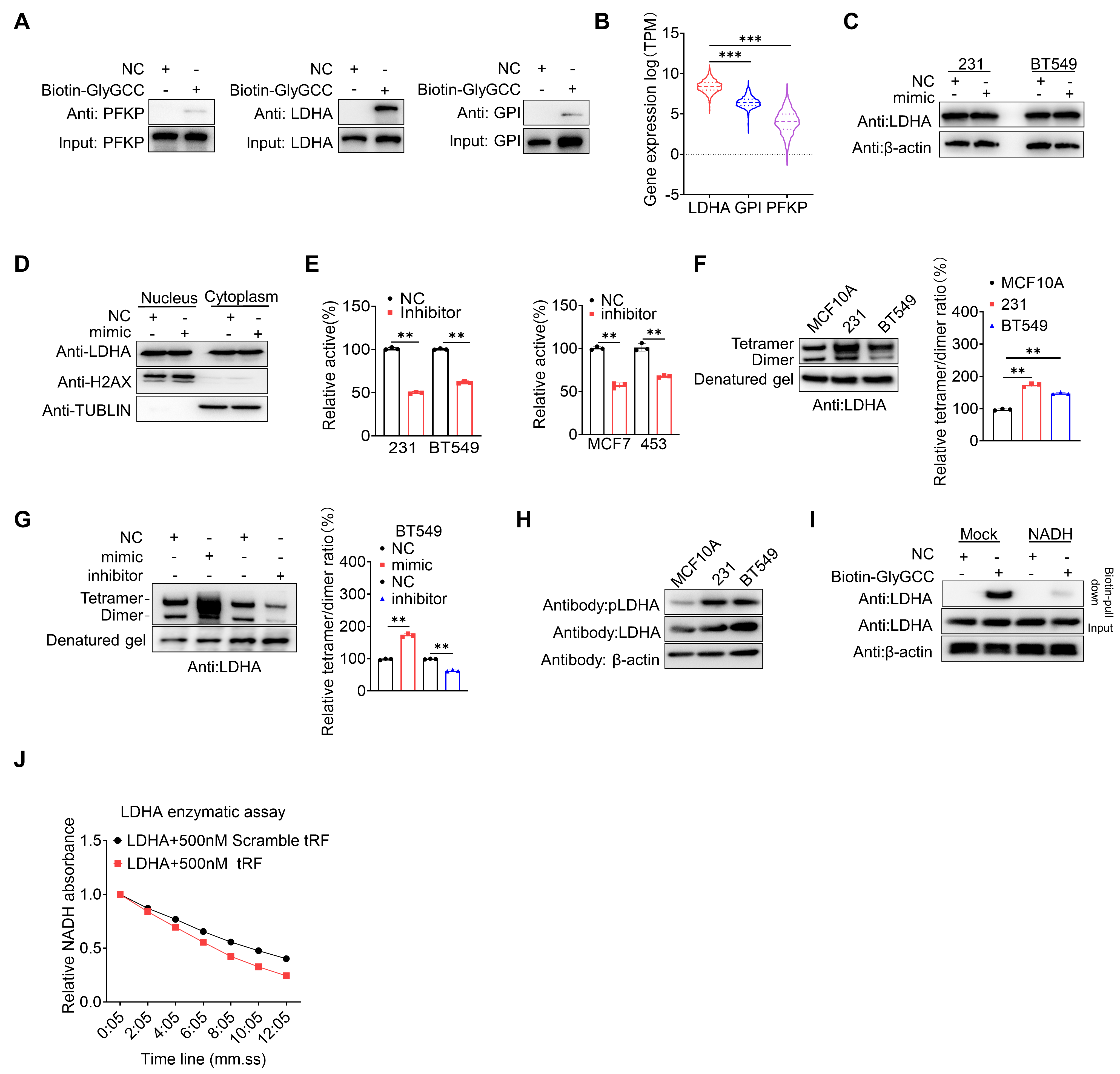


**Figure S2**. **5’tRF-GlyGCC Drives BC Malignancy by Binding LDHA and Boosting its Activity**

A. Western blot analysis was performed to assess the binding capacity of PFKP, LDHA, and GPI to 5’tRF-GlyGCC, derived from RNA pull-down assays utilizing 5’tRF-GlyGCC or a non-targeting oligonucleotide in BC cells.

B. An analysis of the expression profiles of LDHA, GPI, and PFKP in breast tumor tissues was conducted using the TCGA database.

C-D. Western blot analysis was employed to assess the expression levels (C) and distribution (D) of LDHA protein in BC cell lines following transfection with a 5’tRF-GlyGCC mimic.

E. The activity of endogenous LDHA protein was analyzed in BC cells after transfection with 5’tRF-GlyGCC inhibitor.

F. Native Western blot experiments were conducted to evaluate (left panel) and quantitatively determine (right panel) the tetramerization status of LDHA in the breast epithelial cell line MCF-10A, as well as in the BC cell lines MDA-MB-231 and BT549.

G. Native Western blot analysis was conducted to evaluate (left panel) and quantitatively determine (right panel) the effect of transfected 5’tRF-GlyGCC mimic or inhibitor on LDHA tetramer formation in BT549 cells.

H. The Western blot analysis evaluated the phosphorylation status at Y10 of LDHA in breast epithelial cell line MCF10A and BC cell lines MDA-MB-231 and BT549.

I. Pulldown of LDHA from BT549 cell lysate expressing 5’tRF-GlyGCC using Cibacron blue agarose was evaluated by immunoblot.

J. Activity of 500 nM recombinant LDHA measured in the presence of increasing amounts of recombinant 5’tRF-GlyGCC mimic.

Data are presented as mean ± SD from three independent experiments. **P*<0.05, ***P*<0.01, ****P*<0.001, ns, no significant.

**Figure S3**

**
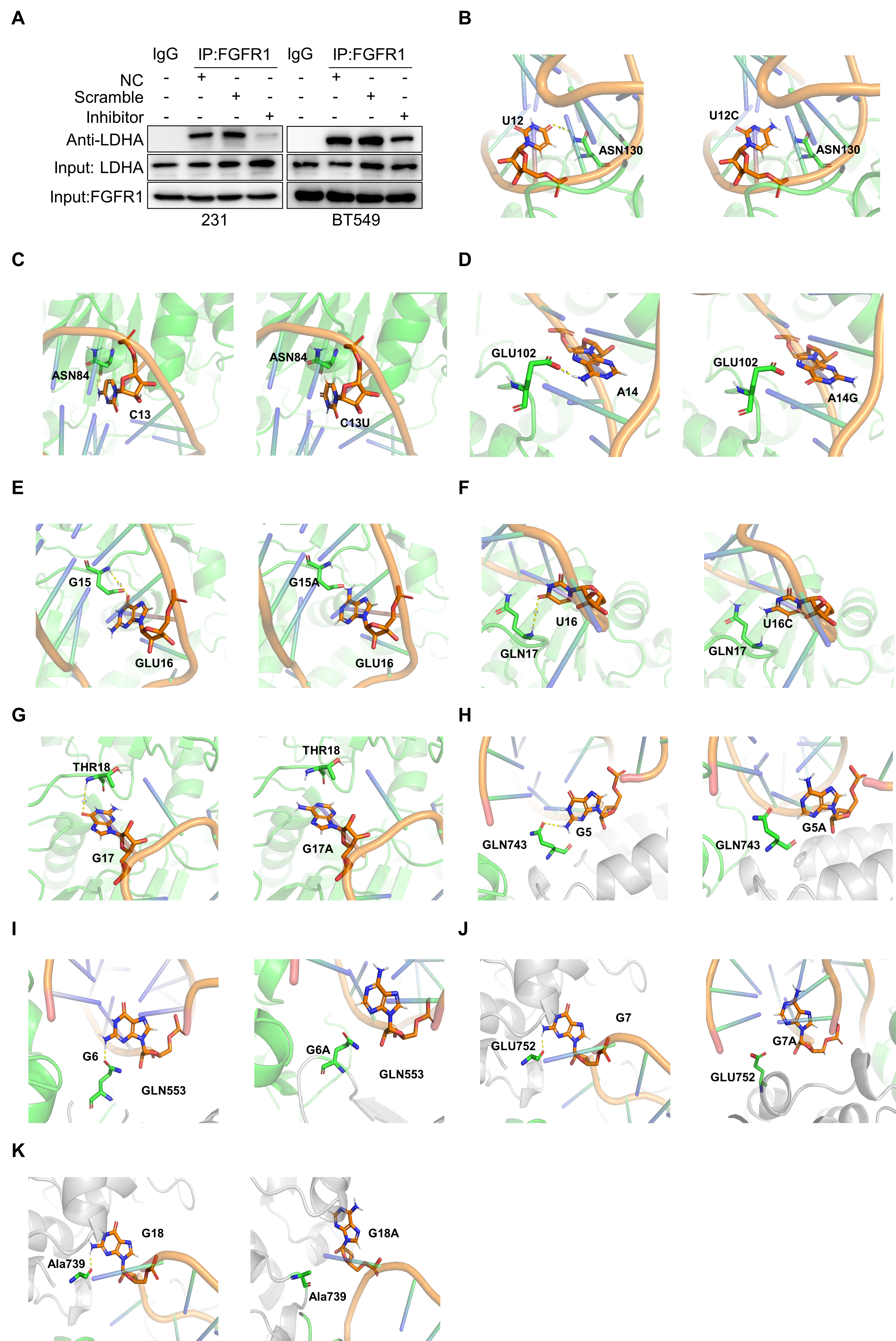
**

**Figure S3. FGFR1 Mediates 5’tRF-GlyGCC-Induced LDHA Activation**

A. MDA-MB-231 (left panel) and BT549 (right panel) cells were subjected to the treatment with negative control (NC), scrambled RNA control, or 5’tRF-GlyGCC inhibitor. The interaction between LDHA and FGFR1 was subsequently analyzed via co-immunoprecipitation (co-IP) assay using an anti-FGFR1 antibody.

B-K. Site-directed mutagenesis analyses of nucleotides predicted by docking models as critical sites for 5'tRF-GlyGCC binding to LDHA and FGFR1. At the LDHA-binding interface: U12 in the wild-type sequence was mutated to C (i.e., U12C) to assess its capacity of forming a hydrogen bond with Asn103 (B), the C13U mutant was used to evaluate the ability of C13 to form hydrogen bonds with Asn84 (C), the A14G mutant was used to examine hydrogen bond formation between A14 and Glu102 (D), the G15A mutant was used to investigate G15’s capacity to form a hydrogen bond with Glu16 (E), the U16C mutant was used to assess the hydrogen bond formation between U16 and Gln17 (F), the G17A mutant was used to evaluate G17’s ability to form hydrogen bonds with Thr18 (G). At the FGFR1-binding interface: the G5A mutation in the wild-type sequence was used to examine hydrogen bond formation between G5 and Gln743 (H), the G6A mutant was used to assess G6’s capacity for hydrogen bonding with Gln553 (I), the G7A mutant was used to evaluate hydrogen bond formation between G7 and Gln752 (J), the G18A mutant was used to investigate G18’s ability to form hydrogen bonds with Ala739 (K). Carbon skeletons of amino acid residues are depicted in green, and those of nucleotide residues in orange. All residues are presented in stick representation.

**Figure S4**


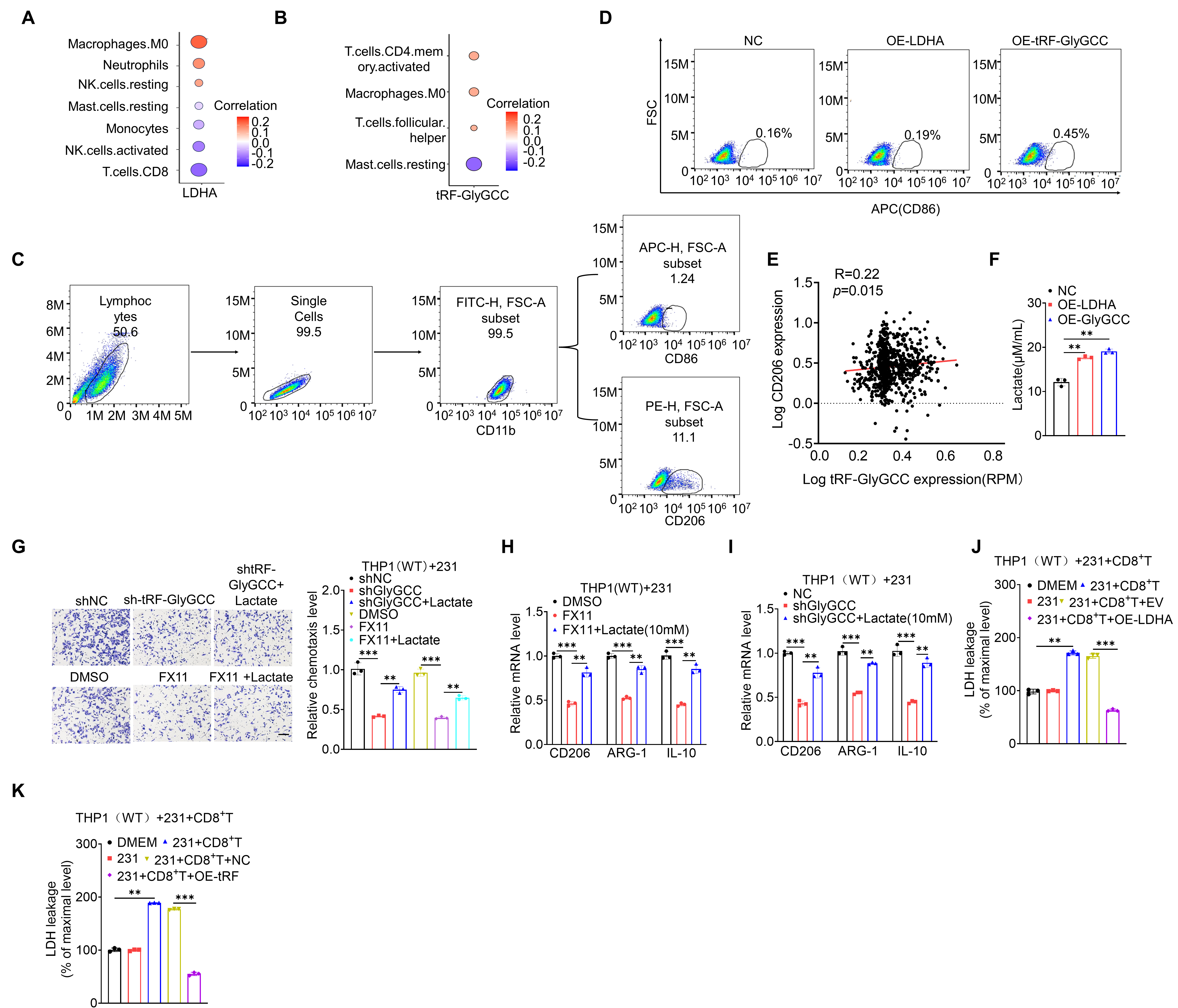


**Figure S4. 5’tRF-GlyGCC /LDHA Induces Infiltration and Polarization of Macrophages**

A-B. Heatmap showing the correlation between (A) LDHA and 5’tRF-GlyGCC (B) gene expression and immune cell infiltration. R > 0.12 and *P* values less than 0.05 were considered to be significant positive correlations, and R < -0.12 and *P* values less than 0.05 were considered to be significant negative correlations. Positive correlations are shown in red, and negative correlations are shown in blue.

C. Illustration of flow cytometry gating strategy for THP1 and RAW cells.

D. Upon stimulating THP1 cells with CM derived from MDA-MB-231 cells stably overexpressing LDHA and 5’tRF-GlyGCC for 48 hours, the alteration in the expression of the M1 macrophage surface marker CD86 was detected using a flow cytometer.

E. Scatter plot analysis demonstrating the relationship between 5’tRF-GlyGCC expression levels and CD206. Correlation is defined by spearman's correlation coefficient. R > 0 indicates positive correlation and R< 0 indicates negative correlation. *P* < 0.05 were considered significant.

F. Lactate concentration assays were conducted to measure lactate levels in MDA-MB-231 cells at 48 h following the stable overexpression of either 5’tRF-GlyGCC or LDHA.

G. Representative images (left panel) and quantification results (right panel) depict the relative migration of THP-1 macrophages, as determined by a transwell assay. The macrophages were stimulated with conditioned medium (CM) collected from MDA-MB-231 cells subjected to the following treatment regimens: (1) expression of shNC; (2) expression of sh-5’tRF-GlyGCC; (3) expression of sh-5’tRF-GlyGCC with supplementation of 10 mM lactic acid; and (4) treatment with or without FX11, followed by the addition of 10 mM lactic acid. All treatments were maintained for 48 h. Scale bar, 100 μm.

H-I. THP-1 cells were stimulated with conditioned medium (CM) derived from MDA-MB-231 cells subjected to either FX11 inhibitor treatment (H) or 5’tRF-GlyGCC knockdown (I), followed by subsequent exposure to 10 mM lactic acid, the expression levels of M2 macrophage markers were quantified via RT-qPCR analysis.

J-K. After stimulating THP1 cells with CM derived from MDA-MB-231 cells stably overexpressing LDHA (J) or 5’tRF-GlyGCC (K), we co-cultured CD8⁺ T cells and MDA-MB-231 cells. The release level of lactate dehydrogenase (LDH) was measured 48 h post-co-culture initiation.

**Figure S5**


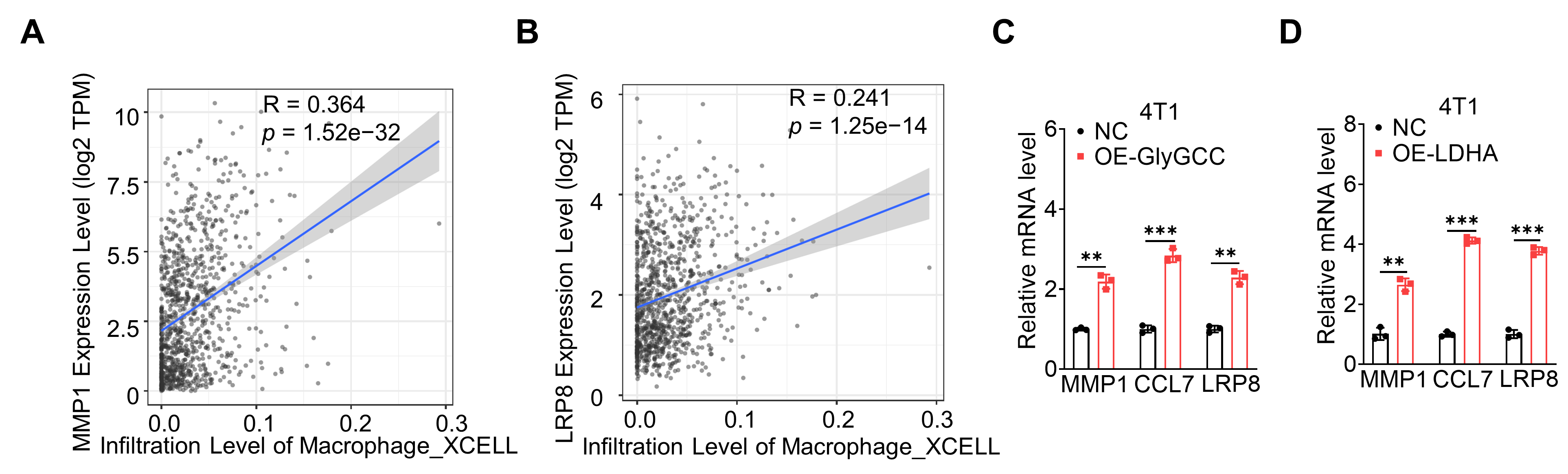


**Figure S5. CCL7 Mediates 5’tRF-GlyGCC/LDHA-driven Macrophage Infiltration and Polarization**

A-B. The scatter plot shows the correlation between MMP1 (A) and LRP8 (B) gene expression and macrophage infiltration levels.

C. qPCR analysis was conducted to evaluate the mRNA levels of MMP1, CCL7, and LRP8 in 4T1 cells stably overexpressing 5’tRF-GlyGCC.

D. qPCR analysis was conducted to evaluate the mRNA levels of MMP1, CCL7, and LRP8 in 4T1 cells stably overexpressing LDHA.

Data are presented as mean ± SD from three independent experiments. **P*<0.05, ***P*<0.01, ****P*<0.001, ns, no significant.

**Figure S6**


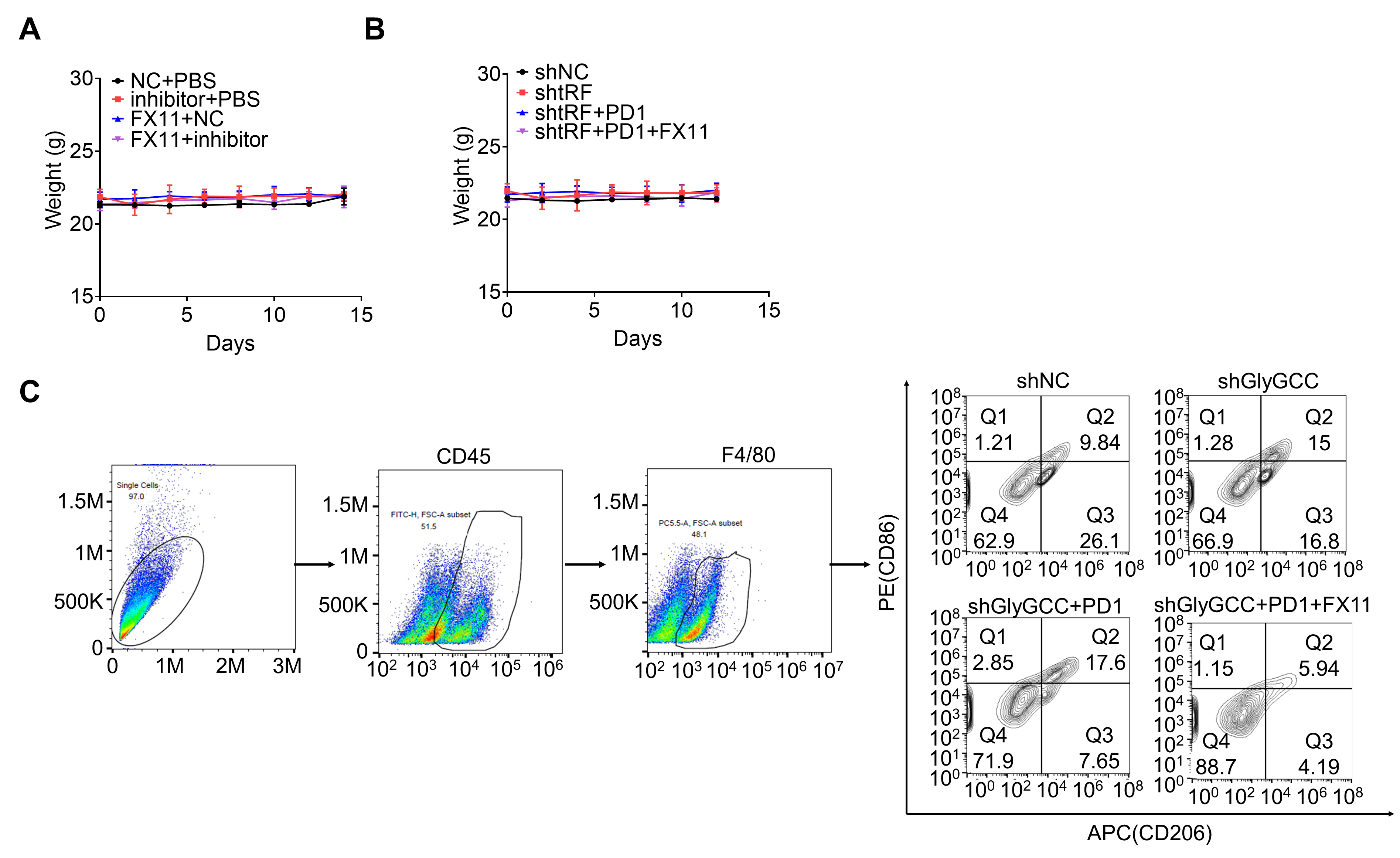


**Figure S6. Targeted Suppress BC Progression Based on 5’tRF-GlyGCC /LDHA Signals**

A**.** The body weight of mice bearing tumors was measured after inoculation with MDA-MB-231 cells. A total of 2×10^5^ cells were injected into the fourth mammary fat pad of Balb/c null mice.

B. The body weight of mice bearing tumors was measured after inoculation with 4T1-shNC, 4T1-sh5’tRF-GlyGCC, 4T1-sh5’tRF-GlyGCC+PD1, and 4T1-5’tRF-GlyGCC+PD1 cells. A total of 1×10^5^ cells were injected into the fourth mammary fat pad of Balb/c mice.

C. Illustration of flow cytometry gating strategy for immune cell analysis in subcutaneous 4T1 tumor transplantation.

**Experimental Section**

1. **Data Mining and Analysis**

Expression analysis of tRF in breast cancer, we downloaded the raw small RNA sequence files ('.bam files') from The Cancer Genome Atlas (TCGA) database with permission from the Data Access Committee. We extracted raw reads (FASTQ format) from the BAM files using the 'bamToFastq' subcommand of the genome computational toolset 'BEDtools2' ^[1]^. Sports 1.1(ref6) software was used to analysis the FASTQ files for tRF identification and abundance. Differential expression analysis of tRFs was calculated using the R package DESeq2 ^[2]^. The fold change greater than 2 or less than -2 with a false discovery rate (FDR) less than 0.05 was considered significant. Survival prognosis analysis, we downloaded the clinical data of the TCGA BRCA cohort from UCSC Xena. Then, the optimal cut-off value of the expression level of 5'tRF-GlyGCC (RPM) was selected for the intergroup threshold. The survival curve is compared by log-rank and the hazard ratio (HR) is displayed. A log-rank *P* < 0.05 was considered statistically significant. Gene Ontology analysis, Gene Ontology (GO) and KEGG analyses were performed using the R package clusterProfiler v.4.10.0 ^[3]^. Terms with a FDR < 0.05 were considered significantly enriched. Calculation of IFN-stimulated gene (ISG) signatures, a previously established method was used to calculate the ISG score derived from the expression profile of a 38-gene signature ^[4]^, the expression levels of 5'tRF-GlyGCC (RPM) were sorted from high to low and divided into high expression group and low expression group according to the lower quartile of expression level. *P* values are determined by two-side Student’s t-test. Signature score calculation, the mean of log2 (TPM + 1) of macrophage marker genes was used as the signature score in the TCGA-BRCA cohort ^[5]^. The expression levels of 5'tRF-GlyGCC (RPM) were sorted from high to low and divided into high expression group and low expression group according to the lower quartile of expression level. Correlations between 5'tRF-GlyGCC expression and signature scores were calculated using the R packages "cor. Test" package with spearman correlation coefficient. *P* values < 0.05 were considered significant.

Tumor Microenvironment Correlation Analysis, the "ESTIMATE algorithm" was performed to analysis the correlation between gene expression and TME by calculating the stromal score and immune score in the TCGA BRCA cohort ^[6]^. The association between TME and LDHA expression was tested using the R packages "cor. Test" with spearman correlation coefficient. R represents the correlation coefficient determined by spearman correlation coefficient, where R > 0 indicates a positive correlation and R < 0 indicates a negative correlation. *P* < 0.05 is defined as statistically significant. Analysis of tumor immune cell infiltration, breast cancer gene expression matrix has been downloaded from TCGA. The LM22 dataset was downloaded from the CIBERSORT database. The CIBERSORT ^[7]^ R package was used to estimate the relative proportions of each cell type of interest. The association between TME and LDHA/5'tRF-GlyGCC expression was determined by spearman's correlation. R > 0 indicates a positive correlation and R < 0 indicates a negative correlation. *P* < 0.05 is defined as statistically significant. Data analysis and visualization, data analysis was conducted using R (v.4.3.3). Data visualization was conducted using ggplot2 (v.3.5.1).

1. **Cell Lines and Cell Culture**

The BC cell lines, including MDA-MB-231 and BT549 cells, as well as the normal breast epithelial cell line MCF10A and human kidney epithelial cell line 293T, were all purchased from the American Type Culture Collection (ATCC, Manassas, VA). Prior to experiments, all cell lines were authenticated using short tandem repeat (STR) profiling (Shanghai Biowing Applied Biotechnology Co., Ltd.) and tested for mycoplasma contamination using the Myco-Blue Mycoplasma Detector (Vazyme), with negative results. All cell lines in our laboratory were cultured in Dulbecco's modified Eagle's medium (DMEM, GIBCO, Carlsbad, CA, USA), supplemented with 10% fetal bovine serum and 1% penicillin–streptomycin (Invitrogen, 100 µg·mL^-1^) additives. The cells were maintained in a humidified atmosphere at 37°C with 5% CO_2_.

1. **Northern Blot Assays**

Total RNA was extracted using Trizol (Invitrogen), then 15 µg of total RNA was subjected to 15% denaturing polyacrylamide gel electrophoresis. The RNA was then transferred to a Biodyne nylon membrane (Pall) using electroporation at 20V for 60 minutes. Following prehybridization for 30 minutes, the membrane was hybridized overnight at 68°C with a denatured 5’tRF-GlyGCC probe (5’-GGC GAG AAT TCT ACC ACT GAA CCA CCC ATG C-3’) labeled with Biotin. After hybridization, the membrane was incubated with an anti-Biotin antibody and the signal on the membrane was detected using Western Blotting Plus Chemiluminescence Reagent (Cat: 34580, Thermo Fisher Scientific).

1. **RNA Extraction and Real-time PCR for Gene Expression**

After extraction, the real-time fluorescent quantitative PCR was performed according to the procedures outlined in our previous study ^[8]^. Primers for targeted genes were as follow:

Human-GAPDH, forward 5’-GCA CCG TCA AGG CTG AGA AC-3’ and reverse 5’-TGG TGA AGA CGC CAG TGGA-3’.

Mouse-GAPDH, forward 5’- CAT CAC TGC CAC CCA GAA GAC TG -3’ and reverse 5’- ATG CCA GTG AGC TTC CCG TTC AG -3’.

5’tRF-GlyGCC, forward 5’-GGG TGG TTC AGT GGT AGA-3’ and reverse 5’- AGT GCA GGG TCC GAG GTA TT-3’.

U6, forward 5’-CTC GCT TCG GCA GCA CA-3’ and reverse 5’-AAC GCT TCA CGA ATT TGC GT -3’.

CCL7, forward 5’-TTGCTCAGCCAGTTGGGATTA-3’ and reverse 5’-AGT CCT GGA CCC ACT TCT GT-3’.

MMP1, forward 5’-GCA CAA ATC CCT TCT ACC CG-3’ and reverse 5’-TGA ACA GCC CAG TAC TTA TTC C-3’.

LRP8, forward 5’-TGT GAG TGC TAC CCT GGC TAC GA-3’and reverse 5’-GCC TTG TCC ATG TAG GCG CTA TAG-3’.

AGO2, forward 5’-GGC CGA CAC AGA TAT TCC A-3’ and reverse 5’- TAA AAT TTA AAC CAC CCC GCA GA-3’.

ALKBH3, forward 5’-AGC CAC GAG TGA TTG ACA GAG-3’ and reverse 5’- ACA AAC AGA CCC TAG ATA CAC CT-3’.

ANG, forward 5’-CTG GGC GTT TTG TTG TTG GTC-3’ and reverse 5’- GGT TTG GCA TCA TAG TGC TGG-3’.

Human-CD206, forward 5’-AGC CAA CAC CAG CTC CTC AAG A-3’ and reverse 5’- CAA AAC GCT CGC GCA TTG TCC A-3’.

Mouse-CD206, forward 5’-GTT CAC CTG GAG TGA TGG TTC TC-3’ and reverse 5’- AGG ACA TGC CAG GGT CAC CTT T-3’.

Human-ARG-1, forward 5’-TCA TCT GGG TGG ATG CTC ACA C-3’ and reverse 5’- GAG AAT CCT GGC ACA TCG GGA A-3’.

Mouse -ARG-1, forward 5’-CAT TGG CTT GCG AGA CGT AGA C -3’ and reverse 5’- GCT GAA GGT CTC TTC CAT CAC C -3’.

Human-IL-10, forward 5’-TCT CCG AGA TGC CTT CAG CAG A-3’ and reverse 5’- TCA GAC AAG GCT TGG CAA CCC A-3’.

Mouse-IL-10, forward 5’- CGG GAA GAC AAT AAC TGC ACC C -3’ and reverse 5’- CGG TTA GCA GTA TGT TGT CCA GC-3’.

Transcription levels of target genes were quantified using threshold cycle numbers. For normalization, the transcripts of the housekeeping gene GAPDH were used under the same culture conditions.

1. **Metabolic Assay**

Glucose and lactate concentrations in cells were measured using a commercial kit (BioVision) according to the manufacturer’s instructions and our previous studies ^[9]^. The quantification of adenosine triphosphate (ATP) was performed using the CellTiter-Glo® luminescent assay (Promega) as per the manufacturer’s guidelines and our earlier research (80). All samples were tested in triplicate. Following a 24 hour transfection of the *in vitro* synthesized 5’tRF-GlyGCC mimic and inhibitor at a concentration of 50 nM into cells, the changes in glucose, ATP, and lactate concentrations were examined.

1. **Extracellular Acidification Rate (ECAR)**

ECAR analysis was conducted using an XF96 Extracellular Flux Analyzer (Seahorse Bioscience), with the procedures as previously described ^[9]^. We transfected BC cells with in *vitro* synthesized 5’tRF-GlyGCC mimic and inhibitor at a concentration of 50 nM. Twenty-four hours later, cells were seeded in unbuffered DMEM supplemented with 10 mM glucose. Basal measurements were obtained under standard conditions, followed by the addition of 2 μM oligomycin and 100 mM 2-deoxyglucose (2-DG) for subsequent measurements.

1. **Cell Proliferation Assay**

Cell growth curve assays were primarily conducted in accordance with the manufacturer's guidelines for the CCK8 kit and our previous research ^[10]^. The procedure involved seeding 2000 cells per well in a 96-well plate one day prior to treatment. The cells were then transfected with either the 5’tRF-GlyGCC mimic or inhibitor at a concentration of 50 nM. Subsequently, 10 μL of CCK8 solution was added to each well as required. The plates were incubated at 37°C for 2 hours, after which the absorbance was measured at 450 nm to assess cell growth.

1. **Colony Formation Assay**

The colony formation assay was utilized to evaluate the proliferative capacity of glioblastoma cells in *vitro*. We transfected BC cells with in *vitro* synthesized 5’tRF-GlyGCC mimics and inhibitors at a concentration of 50 nM. The procedure involved seeding 1500 cells per well in a 6-well plate and culturing them for approximately 8 days. Following this, the cells were fixed and stained with 0.5% crystal violet for 1 hour. This experiment was repeated three times to ensure reliability.

1. **Cell Migration and Invasion Assay**

Cell invasion assays were carried out using a 12-well Transwell plate with an 8 μm pore size (Corning), following the protocol established in our previous study ^[11]^. The procedure involved coating the polycarbonate filter membrane with Matrigel matrix (20 mg/ml, BD Biosciences) and incubating it at 37°C for 30 minutes. We transfected BC cells with in *vitro* synthesized 5’tRF-GlyGCC mimics and inhibitors at a concentration of 50 nM. Twenty-four hours later, 5×10⁴ digested cells per well were seeded in the upper chamber, with a total volume of 200 μL of cell culture medium containing 0.1% FBS. Subsequently, 600 μL of culture medium supplemented with 10% FBS was added to the lower chamber to serve as a chemoattractant. Following incubation for the specified duration, the number of invasive cells was quantified using a phase-contrast microscope (with three random fields selected per chamber). A minimum of three independent experiments were performed to validate the invasion assay.

Cell migration assays were conducted using a 12-well Transwell plate with an 8 μm pore size (Corning). We transfected BC cells with in *vitro* synthesized 5’tRF-GlyGCC mimics and inhibitors at a concentration of 50 nM. Twenty-four hours later, 5×10⁴ digested cells per well were seeded in the upper chamber, with a total volume of 200 μL of cell culture medium containing 0.1% FBS. Subsequently, 600 μL of culture medium supplemented with 10% FBS was added to the lower chamber to serve as a chemoattractant. After incubation for the specified duration, the number of migrated cells was quantified using a phase-contrast microscope (with three random fields selected per chamber). At least three independent experiments were performed to validate the migration assay.

1. **Lentivirus Production, Transduction, siRNA and Plasmids Construction**

The over expression and silencing of 5'tRF-GlyGCC were conducted according to the previous study with slight modification ^[12]^. Specifically, for stable overexpression of 5'tRF-GlyGCC, the synthesized sequence was inserted into the pLKD-CMV-2A-Neo-U6-shRNA vector containing the miR-30 scaffold (OBiO Technology). For stable silencing of 5'tRF-GlyGCC, the antisense sequence was cloned into the lentiviral vector with the miR-30 scaffold, pLKD-CMV-2A-Neo-U6-shRNA. The plasmids and their insertions were authenticated by DNA sequencing. These vectors and the lentiviral packaging system (OBiO Technology) were then cotransfected into 293T cells using Lipo2000 (Invitrogen). The resulting lentiviruses were designated as 5'tRF-GlyGCC overexpression or 5'tRF-GlyGCC silencing, respectively. These lentiviruses were used to infect MDA-MB-231 and BT549 cells in the presence of polybrene (Sigma-Aldrich) and selected with puromycin (Sigma-Aldrich). Finally, the levels of 5'tRF-GlyGCC in the cells were quantified by quantitative reverse transcription polymerase chain reaction (qRT-PCR).

For the construction of FLAG-tagged LDHA expression vectors, full-length and truncated LDHA cDNA were subcloned into the pcDNA3.1-3×FLAG vector (OBiO Technology). For the construction of His-tagged LDHA prokaryotic expression vectors, the full-length LDHA cDNA was cloned into the pET28a-HA vector (OBiO Technology). The siRNA sequences are as follows: si-Ago2-1, 5’- CGT CCG TGA ATT TGG AAT CAT-3’; si-Ago2-2, 5’- CAA TCA AAT TAC AGG CCA ATT-3’; si-ANG-1, 5’-GAA TGG AAA CCC TCA CAG AGA-3’; si-ANG-2, 5’- GCA AGT CTT CTT TCC AGG TCA -3’; si-CCL7-1, 5’- GAA AGC CTC TGC AGC ACT TCT -3’; si-CCL7-2, 5’-GGA TTA ATA CTT CAA CTA CCT-3’;si-MMP1-1,5’GGA GTA ATG TCA CAC CTC TGA-3’; si-MMP1-2, 5’-GAT GAA AGG TGG ACC AAC AAT-3’; si-LRP8-1, 5’-GCA GCC AGA UCU GUG UCA ATT-3’ ; si-LRP8-2, 5’-GGA GAA ACU GGA AGC GGA AGA-3’; si-ALKBH3-1, 5’-CGC ACA CTA AAG AAC CGC ATT-3’; si-ALKBH3-2, 5’- GTT CAG AAA GCG TGG TGT TTG-3’.

1. **RNA Immunoprecipitation Assays**

To explore the enrichment of 5’tRF-GlyGCC by full-length, truncated, and mutant forms of LDHA, we employed RNA-binding protein immunoprecipitation (RIP) assays. Briefly, cells were transiently transfected with 2 μg of plasmids encoding flag-tagged full-length, truncated, and mutant LDHA. The cell lysate, containing a proteasome inhibitor, was then incubated with Anti-FLAG® M2 magnetic beads (Sigma, cat. no. M8823) at 4°C for 2 hours. After five washes with immunoprecipitation lysis buffer, the bound antigens were eluted, and the enrichment of 5’tRF-GlyGCC was quantified using quantitative reverse transcription polymerase chain reaction (qRT-PCR).

1. **Molecular Dynamic (MD) and Ensemble Docking Simulations**

The predicted binding sites and interactions of 5'tRF-GlyGCC on LDHA were investigated by molecular dynamic (MD) and ensemble docking simulations. The X-ray crystal structure of the human LDHA (PDB ID: 4zvv) was used as the initial structure for the MD simulation. The protonation states of the ionizable residues were determined at pH7 based on pKa calculations via both PROPKA and H^++^ programs, and the local hydrogen bonding network was taken into account when the two programs disagreed. MD simulations were conducted using the AMBER18 software package, with the Amber14SB force field employed for the protein. The LDHA system was immersed in an explicit aqueous solvent environment, with water molecules represented by the TIP3P model. A cubic box was utilized, ensuring a 12 Å buffer distance between the box walls and the nearest solute atom, and 10 Cl^-^ ions were added to effectively neutralize the charge. The subsequent energy minimizations and equilibration molecular dynamic simulations followed the same state-of-the-art protocol as in our previous studies, and three independent 100-ns production MD simulations were carried out at a temperature of 310 K with different initial velocities. In all MD simulations, the long-range electrostatic interactions were elegantly handled using the Particle Mesh Ewald (PME) method. A cutoff of 10 Å was applied for both van der Waals and short-range PME interactions, and a time step of 1 fs was employed in conjunction with the SHAKE algorithm in AMBER18.

The ensemble docking simulations were conducted to explore the binding sites and interactions of 5'tRF-GlyGCC on LDHA protein. The initial structures of LDHA utilized for docking were derived from MD simulations by capturing 10 frames at an interval of 5ns from the first 50ns of each MD trajectory. And the initial structure of 5'tRF-GlyGCC fragment was predicted by RNAcomposer. The HDOCK software package was used for the ensemble docking of 5'tRF-GlyGCC onto the LDHA protein. The receptor docking site was defined as amino acid residues 1-162 of chain A within the LDHA protein. The binding affinities of the docking conformations were assessed through application of distance-dependent knowledge-based scoring function (ITScore-PP). The docking results with scores greater than 0.85 were selected for further statistical analysis of participation frequency of amino acid and nucleic acid residues in the formation of hydrogen bond or salt bridge interactions between LDHA and 5'tRF-GlyGCC. Finally, the important amino acid and nucleic acid residues on LDHA protein and 5'tRF-GlyGCC respectively were identified from the statistical analysis of the interaction frequencies. The visualization results of the complex structure were rendered using PyMOL 3.0.

1. **Protein Purification**

Purification of LDHA protein was primarily based on the procedures described in a previous study ^[13]^. In brief, the LDHA gene was cloned into the pRSET-A vector (Thermo Fisher). Subsequently, the vector was transformed into Escherichia coli BL21 (DE3) cells, and the resulting monoclonal clones were sequenced to confirm the correct integration. Following confirmation, monoclonal clones were cultured in LB medium supplemented with 50 mg/L ampicillin at 37°C until the OD600 reached 0.6. Addition of 1 mM IPTG was then made to induce expression, and the culture was continued overnight. Cells were collected by centrifugation and lysed using a fresh lysis buffer without detergents (20 mM Tris-HCl pH 7.4, 100 mM NaCl, 1 mM MgCl_2_, protease inhibitor cocktail (Millipore-Sigma), and PhosSTOP (Millipore-Sigma), followed by sonication. Triton X-100 was added to a final concentration of 1% in the lysate, and the insoluble fraction was removed by centrifugation. The supernatant was assessed for expression levels by immunoblotting. Purification was carried out using two consecutive Ni-NTA agarose (Qiagen) steps. The lysate was incubated with Ni-NTA agarose at 4°C for 2 hours, washed twice with a lysis buffer containing 50 mM imidazole, and then eluted with a lysis buffer containing 500 mM imidazole. The purity of the purified protein was confirmed by SDS-PAGE and Coomassie blue staining, and it was subsequently used for analysis.

1. **Macrophage Migration Assay**

Macrophage migration was evaluated using Boyden chamber inserts (5 μm, Corning) as previously described ^[14]^. Briefly, after co-culturing the cell culture medium of MDA-MB-231 and 4T1 cells stably overexpressing 5’tRF-GlyGCC and LDHA with THP1 and RAW cells for 48 hours, 5×10⁴ digested cells per well were seeded in the upper chamber, with a total volume of 200 μL of cell culture medium containing 0.1% FBS. Subsequently, 600 μL of culture medium supplemented with 10% FBS was added to the lower chamber to serve as a chemoattractant. In certain experiments, anti-CCL7 neutralizing antibody (2 μg/mL, R&D, MAB282) was directly added to the conditioned media. After incubation for the specified duration, the number of migrated cells was quantified using a phase-contrast microscope (with three random fields selected per chamber). At least three independent experiments were performed to validate the migration assay.

1. **Gain of Tumor-Infiltrating Immunocytes and Flow Cytometry**

The method for isolating tumor-infiltrating immune cells from tumor tissue and the protocol for analyzing cell surface markers such as CD45, CD206, CD86, and F4/80 using a flow cytometer were performed as previously described ^[15]^. For surface staining, the antibodies against CD45(Cat:11-0459-42), CD206(Cat:12-2061-82), CD86 (Cat:17-0862-82), and F4/80 (Cat:45-4801-82) were first fixed and permeabilized and incubated at 4°C for 30 minutes. For the flow cytometry detection method of surface molecules on macrophages in the co-culture system, in brief, after co-culturing the cell culture medium of MDA-MB-231 and 4T1 cells stably overexpressing 5’tRF-GlyGCC and LDHA with THP1 and RAW cells for 48 hours, the cells were digested and incubated with flow cytometry antibodies F4/80, CD86, and CD206 for 30 minutes. Subsequently, the cells were washed twice with PBS, detected by CytoFLEX S, and the data was analyzed using FlowJo V10 software. All flow cytometry antibodies were purchased from Thermo Fisher Scientific Co. Ltd., USA.

1. **Western Blot and Native Polyacrylamide Gel Electrophoresis Analysis**

Western blotting was performed as outlined in our previous study ^[8]^. In brief, we transfected BC cells with in *vitro* synthesized 5’tRF-GlyGCC mimics and inhibitors at a concentration of 50 nM. Twenty-four hours later, cells were lysed in a buffer composed of 50 mM Tris-HCl (pH 7.6), 150 mM NaCl, 1 mM EDTA, 1% NP-40, 0.5% sodium deoxycholate, 5 mg/ml aprotinin, 5 mg/ml leupeptin, and 1 mM phenylmethylsulfonyl fluoride, and the lysis was conducted at 4°C for 30 minutes. Thereafter, 20 μg of total protein was separated using sodium dodecyl sulfate-polyacrylamide gel electrophoresis (SDS-PAGE) and transferred to a polyvinylidene fluoride (PVDF) membrane. The membrane was blocked with 5% non-fat milk at room temperature for 2 hours before overnight incubation with primary antibodies (LDHA, Proteintech, 19987-1-AP; ALKBH3, Merck Millipore, 09-882; Phospho-LDHA (Tyr10), CST, #8176; ANG, Proteintech, 18302-1-AP; β-actin, CST, #4970; Flag, Sigma, F1804; Biotin HRP-linked Antibody, CST #7075) at 4°C. After three washes, the membrane was incubated with horseradish peroxidase (HRP)-conjugated secondary antibodies (1:5,000 dilution) for 2 hours at room temperature, and the Western blot signals were visualized using Western Blotting Plus Chemiluminescence Reagent (Cat: 34580, Thermo Fisher Scientific). The protein extraction process and 5×sample loading buffer preparation were identical to those described previously. The samples were then loaded onto a Criterion Tris-HCl protein gel (Bio-Rad) without SDS and subjected to electrophoresis. Following electrophoresis, the proteins were transferred to a PVDF membrane using the previously described methods for Western blotting detection.

1. **LDH Activity Assay**

LDH activity was quantified per the manufacturer's instructions (MAK066, Sigma-Aldrich). Briefly, 25 ng of recombinant LDHA was pre-incubated with a synthetic 5’tRF-GlyGCC mimic (2 µg) on ice for 30 minutes. Samples were then transferred to a 96-well plate and supplemented with the substrate mix. Absorbance readings at 450 nm were obtained every 2 minutes until saturation was achieved. For lysate assays, LDHA protein was immunoprecipitated from breast cancer cells with 5'tRF-GlyGCC overexpression, and subsequently added to a reaction buffer composed of 0.2 M Tris-HCl (pH 7.3), 0.05% bovine serum albumin, 10 mM magnesium chloride, 2 mM pyruvate, and 20 μM NADH. A spectrophotometer was utilized to monitor absorbance changes at 340 nm; the reduction in absorbance directly reflects the oxidation rate of NADH, which was further used to quantify the enzymatic activity of LDHA.

1. **Mass spectrometry**

The LC-MS/MS assay was conducted as previously described. In essence, a biotin-labeled 5’tRF-GlyGCC mimic (2 µg), synthesized in *vitro*, was incubated with MDA-MB-231 cell lysate, followed by a Biotin pull-down analysis as previously detailed ^[16]^. The Biotin pull-down samples were then subjected to Coomassie Brilliant Blue staining for analysis. Subsequently, all protein bands were excised and digested overnight at 37°C with trypsin, including reduction with DTT and alkylation with iodoacetamide. The digested samples were concentrated to near dryness and desalted using C18 stage tips. The desalted samples were then coupled online to a Thermo Fisher Scientific EASY-nLC 1000 system and a Fusion Lumos mass spectrometer (Thermo Fisher Scientific) for peptide analysis. The raw data were processed using MaxQuant version 1.5.5.1 (Max Planck Institute) for protein identification.

1. **Immunohistochemistry (IHC)**

Immunohistochemistry was employed to assess the expression of the target protein, following the procedures outlined in our previous research ^[11]^.

**Reference**

[1] A. R. Quinlan, I. M. Hall, “BEDTools: a flexible suite of utilities for comparing genomic features”, *Bioinformatics (Oxford, England)* **2010**, *26* (6), 841, <https://doi.org/10.1093/bioinformatics/btq033>.

[2] S. Anders, W. Huber, “Differential expression analysis for sequence count data”, *Genome Biology* **2010**, *11* (10), R106, <https://doi.org/10.1186/gb-2010-11-10-r106>.

[3] T. Wu, E. Hu, S. Xu, M. Chen, P. Guo, Z. Dai, T. Feng, L. Zhou, W. Tang, L. Zhan, X. Fu, S. Liu, X. Bo, G. Yu, “clusterProfiler 4.0: A universal enrichment tool for interpreting omics data”, *Innovation (Cambridge (Mass.))* **2021**, *2* (3), 100141, <https://doi.org/10.1016/j.xinn.2021.100141>.

[4] H. Liu, J. Golji, L. K. Brodeur, F. S. Chung, J. T. Chen, R. S. deBeaumont, C. P. Bullock, M. D. Jones, G. Kerr, L. Li, D. P. Rakiec, M. R. Schlabach, S. Sovath, J. D. Growney, R. A. Pagliarini, D. A. Ruddy, K. D. MacIsaac, J. M. Korn, E. R. McDonald, 3rd, “Tumor-derived IFN triggers chronic pathway agonism and sensitivity to ADAR loss”, *Nature Medicine* **2019**, *25* (1), 95, <https://doi.org/10.1038/s41591-018-0302-5>.

[5] Z. Tang, B. Kang, C. Li, T. Chen, Z. Zhang, “GEPIA2: an enhanced web server for large-scale expression profiling and interactive analysis”, *Nucleic Acids Research* **2019**, *47* (W1), W556, <https://doi.org/10.1093/nar/gkz430>.

[6] T. Li, J. Fu, Z. Zeng, D. Cohen, J. Li, Q. Chen, B. Li, X. S. Liu, “TIMER2.0 for analysis of tumor-infiltrating immune cells”, *Nucleic Acids Research* **2020**, *48* (W1), W509, <https://doi.org/10.1093/nar/gkaa407>.

[7] A. M. Newman, C. L. Liu, M. R. Green, A. J. Gentles, W. Feng, Y. Xu, C. D. Hoang, M. Diehn, A. A. Alizadeh, “Robust enumeration of cell subsets from tissue expression profiles”, *Nature Methods* **2015**, *12* (5), 453, <https://doi.org/10.1038/nmeth.3337>.

[8] Z. Chen, M. Qi, B. Shen, G. Luo, Y. Wu, J. Li, Z. Lu, Z. Zheng, Q. Dai, H. Wang, “Transfer RNA demethylase ALKBH3 promotes cancer progression via induction of tRNA-derived small RNAs”, *Nucleic Acids Research* **2019**, *47* (5), 2533, <https://doi.org/10.1093/nar/gky1250>.

[9] Y. Wu, Z. Chen, G. Xie, H. Zhang, Z. Wang, J. Zhou, F. Chen, J. Li, L. Chen, H. Niu, H. Wang, “RNA m(1)A methylation regulates glycolysis of cancer cells through modulating ATP5D”, *Proceedings of the National Academy of Sciences of the United States of America* **2022**, *119* (28), e2119038119, <https://doi.org/10.1073/pnas.2119038119>.

[10] Q. Liu, Z. Chen, G. Jiang, Y. Zhou, X. Yang, H. Huang, H. Liu, J. Du, H. Wang, “Epigenetic down regulation of G protein-coupled estrogen receptor (GPER) functions as a tumor suppressor in colorectal cancer”, *Molecular Cancer* **2017**, *16* (1), 87, <https://doi.org/10.1186/s12943-017-0654-3>.

[11] Z. J. Chen, W. Wei, G. M. Jiang, H. Liu, W. D. Wei, X. Yang, Y. M. Wu, H. Liu, C. K. Wong, J. Du, H. S. Wang, “Activation of GPER suppresses epithelial mesenchymal transition of triple negative breast cancer cells via NF-κB signals”, *Molecular Oncology* **2016**, *10* (6), 775, <https://doi.org/10.1016/j.molonc.2016.01.002>.

[12] Z. Su, C. Kuscu, A. Malik, E. Shibata, A. Dutta, “Angiogenin generates specific stress-induced tRNA halves and is not involved in tRF-3-mediated gene silencing”, *The Journal of Biological Chemistry* **2019**, *294* (45), 16930, <https://doi.org/10.1074/jbc.RA119.009272>.

[13] M. R. Woodford, A. J. Baker-Williams, R. A. Sager, S. J. Backe, A. R. Blanden, F. Hashmi, P. Kancherla, A. Gori, D. R. Loiselle, M. Castelli, S. A. Serapian, G. Colombo, T. A. Haystead, S. M. Jensen, W. G. Stetler-Stevenson, S. N. Loh, L. S. Schmidt, W. M. Linehan, A. Bah, D. Bourboulia, G. Bratslavsky, M. Mollapour, “The tumor suppressor folliculin inhibits lactate dehydrogenase A and regulates the Warburg effect”, *Nature Structural & Molecular Biology* **2021**, *28* (8), 662, <https://doi.org/10.1038/s41594-021-00633-2>.

[14] M. Kong, W. Dong, Y. Zhu, Z. Fan, X. Miao, Y. Guo, C. Li, Y. Duan, Y. Lu, Z. Li, Y. Xu, “Redox-sensitive activation of CCL7 by BRG1 in hepatocytes during liver injury”, *Redox Biology* **2021**, *46*, 102079, <https://doi.org/10.1016/j.redox.2021.102079>.

[15] X. Dai, L. Lu, S. Deng, J. Meng, C. Wan, J. Huang, Y. Sun, Y. Hu, B. Wu, G. Wu, J. F. Lovell, H. Jin, K. Yang, “USP7 targeting modulates anti-tumor immune response by reprogramming Tumor-associated Macrophages in Lung Cancer”, *Theranostics* **2020**, *10* (20), 9332, <https://doi.org/10.7150/thno.47137>.

[16] L. Pan, X. Huang, Z. X. Liu, Y. Ye, R. Li, J. Zhang, G. Wu, R. Bai, L. Zhuang, L. Wei, M. Li, Y. Zheng, J. Su, J. Deng, S. Deng, L. Zeng, S. Zhang, C. Wu, X. Che, C. Wang, R. Chen, D. Lin, J. Zheng, “Inflammatory cytokine-regulated tRNA-derived fragment tRF-21 suppresses pancreatic ductal adenocarcinoma progression”, *The Journal of Clinical Investigation* **2021**, *131* (22), <https://doi.org/10.1172/jci148130>.
